# Machine learning algorithms’ accuracy in predicting kidney disease progression: a systematic review and meta-analysis

**DOI:** 10.1186/s12911-022-01951-1

**Published:** 2022-08-01

**Authors:** Nuo Lei, Xianlong Zhang, Mengting Wei, Beini Lao, Xueyi Xu, Min Zhang, Huifen Chen, Yanmin Xu, Bingqing Xia, Dingjun Zhang, Chendi Dong, Lizhe Fu, Fang Tang, Yifan Wu

**Affiliations:** 1 The Second Clinical Medical College of Guangzhou University of Chinese Medicine, Guangzhou, China; 2 Department of Nephrology, Guangdong Provincial Hospital of Chinese Medicine, The Second Affiliated Hospital of Guangzhou University of Chinese Medicine, Guangzhou, China; 3 Chronic Disease Management Department, Guangdong Provincial Hospital of Chinese Medicine, The Second Affiliated Hospital of Guangzhou University of Chinese Medicine, Guangzhou, China

**Keywords:** Artificial intelligence, Machine learning algorithm, Prediction models, Chronic kidney disease, CKD progression, Immunoglobulin A nephropathy

## Abstract

**Background:**

Kidney disease progression rates vary among patients. Rapid and accurate prediction of kidney disease outcomes is crucial for disease management. In recent years, various prediction models using Machine Learning (ML) algorithms have been established in nephrology. However, their accuracy have been inconsistent. Therefore, we conducted a systematic review and meta-analysis to investigate the diagnostic accuracy of ML algorithms for kidney disease progression.

**Methods:**

We searched PubMed, EMBASE, Cochrane Central Register of Controlled Trials, the Chinese Biomedicine Literature Database, Chinese National Knowledge Infrastructure, Wanfang Database, and the VIP Database for diagnostic studies on ML algorithms’ accuracy in predicting kidney disease prognosis, from the establishment of these databases until October 2020. Two investigators independently evaluate study quality by QUADAS-2 tool and extracted data from single ML algorithm for data synthesis using the bivariate model and the hierarchical summary receiver operating characteristic (HSROC) model.

**Results:**

Fifteen studies were left after screening, only 6 studies were eligible for data synthesis. The sample size of these 6 studies was 12,534, and the kidney disease types could be divided into chronic kidney disease (CKD) and Immunoglobulin A Nephropathy, with 5 articles using end-stage renal diseases occurrence as the primary outcome. The main results indicated that the area under curve (AUC) of the HSROC was 0.87 (0.84–0.90) and ML algorithm exhibited a strong specificity, 95% confidence interval and heterogeneity (I^2^) of (0.87, 0.84–0.90, [I^2^ 99.0%]) and a weak sensitivity of (0.68, 0.58–0.77, [I^2^ 99.7%]) in predicting kidney disease deterioration. And the the results of subgroup analysis indicated that ML algorithm’s AUC for predicting CKD prognosis was 0.82 (0.79–0.85), with the pool sensitivity of (0.64, 0.49–0.77, [I^2^ 99.20%]) and pool specificity of (0.84, 0.74–0.91, [I^2^ 99.84%]). The ML algorithm’s AUC for predicting IgA nephropathy prognosis was 0.78 (0.74–0.81), with the pool sensitivity of (0.74, 0.71–0.77, [I^2^ 7.10%]) and pool specificity of (0.93, 0.91–0.95, [I^2^ 83.92%]).

**Conclusion:**

Taking advantage of big data, ML algorithm-based prediction models have high accuracy in predicting kidney disease progression, we recommend ML algorithms as an auxiliary tool for clinicians to determine proper treatment and disease management strategies.

**Supplementary Information:**

The online version contains supplementary material available at 10.1186/s12911-022-01951-1.

## Background

Chronic kidney disease (CKD) affects 8–16% of the world’s population, and has become a global public health problem as its prevalence has increased [1, 2]. As the 10th leading cause of death in the world [3], 1.2 million people died from CKD in 2017 globally [4]. Kidney injury is an irreversible process, any form of kidney disease can progress to end-stage renal diseases (ESRD), and may require Renal Replacement Therapy (RRT) for residual renal function damage [5, 6]. Patients who finally progress to ESRD or undertake RRT suffer from heavy economic pressure [7]. However, observational studies have shown that the speed and severity of kidney disease progression varies [8, 9]. Therefore, early identification of groups at high-risk of kidney disease progression accurately and delaying kidney function deterioration, have become an important focus in kidney disease management [10–12].

Considering the increasing prevalence and variegated severity of disease progression, kidney disease patients must be managed through stratification. An accurate disease prognosis prediction model may assist medical staff in early intervention for high-risk patients with poor prognosis. Management strategies should be adopted based on the predictable outcome. In order to promote early identification of patients at high risk of kidney function deterioration, researchers have conducted numerous studies exploring the risk factors, and have established several risk prediction models [13, 14].

These models have performed well in internal validation, but their capacity for generalization is uncertain because only a portion of the studies have been externally validated. As a new tool for big data analysis, machine learning (ML) has emerged in the field of medicine in recent years [15]. ML allows the construction of an algorithm that can learn, predict, and improve with experience [16] based on big data. It has immense potential in exploring risk factors for disease progression and predicting patients’ prognosis. Several ML algorithm-based prediction models have been successful in predicting kidney function during a specific period of time, and shown greater capacity for generalization than previous statistical models.

Though ML algorithms can extract meaningful patterns from big data, several problems remain in clinical practice. Firstly, selecting suitable models in clinical practice is challenging, due to the lack of evidence. Because previous systematic reviews pertaining to prognostic prediction models have neither focused on ML algorithms, nor have they extracted data for further analysis. Secondly, researchers have used an array of ML algorithms, predictors, and outcome indicators to construct prediction models. Finally, there is a dearth of high quality research demonstrating the accuracy and reliability of ML algorithm-based prediction models. Thus, experienced clinicians rely more on their own knowledge and experience when judging patients’ prognosis.

Considering the potentiality and problems pertaining to ML algorithms in the field of nephrology, there is a need for a summary of current research on ML algorithm-based prognostic prediction models for the deterioration of various kidney diseases. Therefore, we conducted this systematic review by reviewing relevant studies and extracting data for a meta-analysis. In doing so, we investigated ML algorithms’ accuracy in predicting kidney disease progression.

## Methods

The methods and results of this review are presented according to the Preferred Reporting Items for Systematic reviews and Meta-analyses statement (PRISMA). The review protocol was previously registered on PROSPERO (International Prospective Register of Systematic Reviews) with the CRD (Centre of Reviews and Dissemination), report number CRD42020156213.

### Eligibility criteria

The inclusion criteria were:

1. Clinical studies of diagnostic tests of accuracy.
2. Participants with kidney disease, aged 18 years or older.
3. Studies that used ML algorithms.
4. Study outcome reflected kidney disease deterioration, including the doubling of serum creatinine, sudden estimated Glomerular Filtration Rate (eGFR) deterioration, urinary protein level aggravation, ESRD occurrence, RRT initiation, cardiovascular events and all-cause mortality.
5. Studies from which indicators could be extracted that pertained to diagnostic test accuracy, such as accuracy, specificity, sensitivity, true positive (TP), false positive (FP), true negative (TN), false negative (FN), Area Under Curve(AUC) and C-statistic.
6. Studies published in either English or Chinese.

### Search strategy

We searched PubMed, EMBASE, Cochrane Central Register of Controlled Trials, the Chinese Biomedicine Literature Database, Chinese National Knowledge Infrastructure, Wanfang Database, and VIP Database by using both free-text terms and Medical Subject Headings (MeSH) terms for studies limited to humans, without any language restrictions, from the establishment of these databases until October 2020. The detailed search strategies are listed in the Additional file 1. The last search was performed on October 31, 2020.

### Study selection

The records retrieved from the search were imported to NoteExpress 3.2.0. Two authors (M. T. Wei and N. L.) independently screened the records by title and abstract after deduplication. Then, the full texts of the selected records were read independently by two researchers (M. T. Wei and N. L.). At each stage of selection, disagreements were arbitrated by a third reviewer (X. L. Zhang) and resolved by consensus. After that, each researcher created an Excel spreadsheet of the articles to be excluded, and their exclusion reasons, before we compiled a final list of included articles.

### Data extraction

Two independent reviewers (M. T. Wei and N. L.) extracted data using a customized extraction form. Considering an individual article may use several ML algorithms to build prediction models separately or in combination, in order to explicate the performance of a single ML algorithm, we extracted data in the unit of a single ML algorithm rather than a single article. The extracted data included the TP, FP, FN and TN numbers of patients with kidney disease progression predicted by an ML algorithm, and the ML algorithm’s accuracy, sensitivity, specificity, positive predictive value (PPV) and negative predictive value (NPV). During the data extraction process, we used RevMan 5.2 for data conversion.

### Assessment methodology quality

We used the Quality Assessment of Diagnostic Accuracy Studies 2 (QUADAS-2) tool to assess the quality of the included studies. Two reviewers adjusted both the signaling questions and the assessment questions to build a specific version of the tool, according to our study’s objective. We then tested the tool, and when we achieved good agreement, we determined that it would be the final version of the review tool. Both authors then used it to independently assess the risk of bias and the applicability of all included studies. Disagreements were resolved by consensus.

### Data synthesis

Considering the bivariate nature of the data, we used both the bivariate model and the hierarchical summary receiver operating characteristic (HSROC) method for data synthesis. The bivariate model, which preserved the bivariate nature of the data, was used to summarize the index tests’ hierarchical sensitivity and specificity. The HSROC model, which could convert the bivariate data into univariate data, was used to determine the index tests’ overall accuracy. In the absence of covariates, the bivariate model was equivalent to the HSROC model [17].

To judge whether there was a threshold effect between the studies, we used the correlation coefficient between the logit transformed sensitivity and specificity generated by the bivariate model, and the asymmetry parameter β generated by the HSROC model. Where a negative correlation coefficient or β = 0 showed an expected trade-off between sensitivity and specificity across thresholds, test accuracy could be represented by the expected accuracy (logDOR) [18]. Then we used the bivariate model to estimate the pool sensitivity and specificity, and generated a forest plot. After that, we generated the HSROC curves and their 95% prediction intervals via the HSROC model. We also calculated the HSROC’s AUC and diagnostic odds ratios (DORs) to evaluate the overall accuracy.

An I^2^ statistic was used to explore the heterogeneity. There is potential heterogeneity when the I^2^ is greater than 50%. Heterogeneity was also examined visually through HSROC plots. In order to identify any sources of heterogeneity, we conducted a meta regression when there were more than 10 single ML algorithms included. Then, we followed with a subgroup analysis after identifying any sources of heterogeneity. Finally, we conducted sensitivity analysis, and combined the data after eliminating outliers and data with small sample sizes to assess the index test’s stability. We used Deeks' funnel plot asymmetry test to evaluate publication bias. And the data synthesis was conducted with RevMan 5.2 and Stata (version 15.0), using the "Metandi" and "Midas" packages.

## Results

**Fig. 1:**
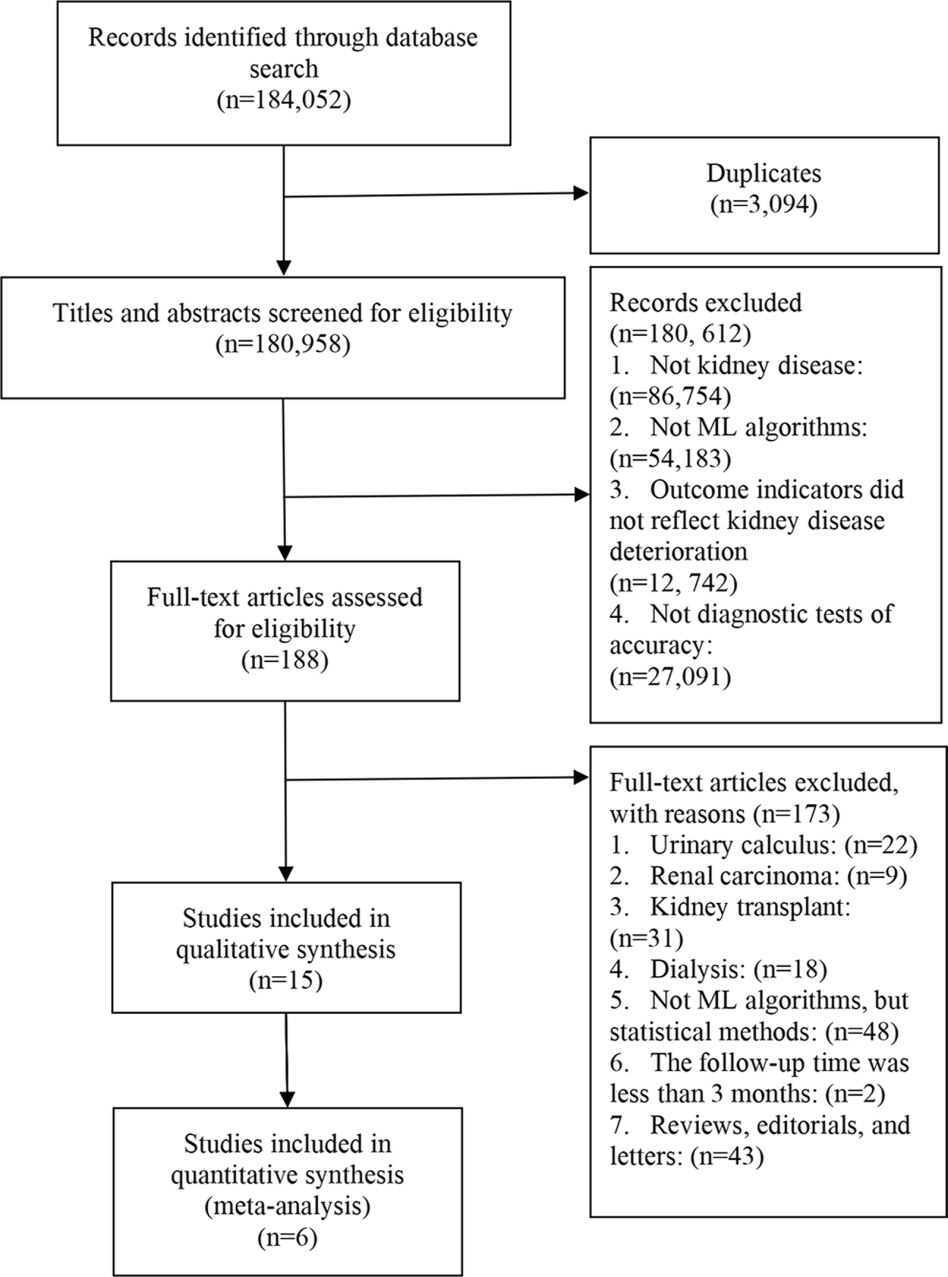
PRISMA flow diagram of study selection process

We retrieved a total of 184,052 articles from the literature databases. After removing duplicates, we screened 180,958 records by title and abstract, and excluded 180,612. Then, we evaluated the full texts of the remaining 188 articles based on the study eligibility criteria. Ultimately, 15 studies were included in our systematic review. However, we were unable to extract the specific TP, FP, FN and TN data from 9 of the articles. Therefore, only 6 articles were eligible for data synthesis. A detailed flow diagram with the study selection process and reasons for exclusion is shown in Fig. 1.

### Clinical application

The total sample size of the 15 articles was 115,155, and the mean age was 59.28 years old. 23 ML algorithms were used to construct progression models with 6 types of predictors—demographics, comorbidities, laboratory data from blood and urine samples, renal biopsy pathology, and therapeutic regimen. These algorithms’ accuracy varied, as did the evaluation indexes used to evaluate the accuracy (see Table 1).

**Table 1:** Clinical characteristics of the included studies

Studies	Country	N	Ages (years)	Men (%)	KD/Outcome	Follow up times(Y)	Reporting dataset	ML algorithm	Optimal model	AUC	Accuracy	C statistic	Analysis	TP	FP	FN	TN
Goto [19], 2009	Japan	790	28.14	41.8	IgAN/doubling of SCR	10.0	Training	DTs	DTs	0.830	N/A	N/A	N/A	N/A	N/A	N/A	N/A
Diciolla [20], 2015	Italy Norway Japan	1040	34.56	69.13	IgAN/ESRD	N/A	Training	ANN NFS SVM DTs	ANN	N/A	90.1	N/A	(1) ANN (2) NFS (3) SVM (4) DTs	177 175 180 178	35 63 97 67	64 66 61 63	764 736 702 732
Pesce [21], 2016	Italy	546	32.5	68.13	IgAN/ESRD	5.0^*^	Testing	ANN	ANN	89.9	91.8	N/A	(1) ANN	14	2	7	87
	Norway	441	38.2	72.56	IgAN/ESRD	6.0^*^	Testing	ANN	ANN	93.3	92.1	N/A	(2) ANN	23	3	4	59
	Japan	53	32.7	50.94	IgAN/ESRD	3.0^*^	Testing	ANN	ANN	100	90.9	N/A	(3) ANN	1	1	0	9
Cheng [22], 2017	Taiwan, China	463	65	N/A	CKD/ESRD	1.5	Training	C4.5 CART SVM Adaboost	CART + Adaboost	0.715	0.662	N/A	(1) C4.5 (2) CART (3) SVM	73 76 80	57 48 45	59 56 52	75 84 87
Feng [23], 2018	China	119	N/A	N/A	CKD/CKD STAGE	N/A	Training	DTs	DTs	N/A	0.9827	N/A	N/A	N/A	N/A	N/A	N/A
Liu [24], 2018	China	262	32.71	47.3	IgAN/ESRD	3.0	Training	RF	RF	0.9729	N/A	N/A	N/A	N/A	N/A	N/A	N/A
Helena [8], 2019	U.S	4640	60.19	N/A	CKD/ESRD	3.7	Testing	Lasso	Lasso	N/A	N/A	0.863	N/A	N/A	N/A	N/A	N/A
Xiao [25], 2019	China	551	58.15	N/A	CKD/PRO	3.0	Testing	LR ElasticN Lasso Ridge SVM RF KNN ANN XGBoost	LR	0.873	0.82	N/A	(1) LR (2) ElasticN (3) Lasso (4) Ridge (5) SVM (6) RF (7) KNN (8) ANN’ (9)XGBoost	54 52 52 51 53 52 49 52 55	8 7 7 8 8 8 12 8 8	12 14 14 14 13 14 17 14 11	37 38 38 37 37 37 33 37 37
Chen [26], 2020	China	2047	34.80	49.25	IgAN/ ESRD	5.0^1^	Training + Testing	LR SVM DTs ANN RF XGBoost	XGBoost	N/A	N/A	0.89	N/A	N/A	N/A	N/A	N/A
Masaki [27], 2020	Japan	30,810	N/A	N/A	DKD/stage of DKD	0..5	Training	LR CNN	LR	0.722	N/A	N/A	N/A	N/A	N/A	N/A	N/A
Dovgan [28], 2020	Taiwan, China	8492	N/A	N/A	CKD/ RRT	1.0	Training	LR XGBoost SGD SVM BDTs ANN RF Bayes DTs NN	LR	0.778	N/A	N/A	(1) LR (2)XGBoost (3) SGD	651 60 152	1628 52 164	394 985 893	5819 7395 7283
Nagaraj [29], 2020	Netherlands	11,789	62.75	N/A	DKD/ESRD	2.7	Testing	LR SVM RF FNN	FNN	0.84	N/A	N/A	N/A	N/A	N/A	N/A	N/A
Schena [30], 2020	Italy	948	40.6	72.2	IgAN/ESRD	7.4	Training	ANN	ANN	0.89	0.83	N/A	(1) ANN	36	8	6	117
Yuan [31], 2020	China	1090	50.01	56.3	CKD/ESRD	4.0	Training	LR RF SVM NNET	RF	0.878	N/A	N/A	N/A	N/A	N/A	N/A	N/A
Zhou (9), 2020	China	2507	70.6	N/A	CKD/ESRD	3.0	Training	LR	LR	N/A	N/A	0.69	N/A	N/A	N/A	N/A	N/A

*CKD* chronic kidney disease, *DKD* diabetes kidney disease, *IgAN* Immunoglobulin A Nephropathy, *ESRD* end stage renal disease, *RRT* renal replace therapy, *PRO* proteinuria, *TP* true positive, *FP* false positive, *FN* false negative, *TN* true negative, *AUC* area under the curve, *DTs* Decision Trees, *ANN* artificial neural network, *NFS* neural fuzzy systems, *SVM* support vector machine, *RF* random forest, *LR* logistic regression, *ElasticN* Elastic Net, *KNN* K Nearest Neighbors, *NN* Nearest Neighbors, *SGD* Stochastic Gradient Descent, *BDTs* Bagging Decision Trees, *CNN* convolutional neural network, *CART* classification and regression, *Lasso* Lasso regression, *Ridge* Ridge regression, *NNET* neural network, *FNN* Feed-forward neural network

*Median times

### Kidney disease types

The various kidney diseases in the included articles could be classified into 3 categories: CKD, Immunoglobulin A Nephropathy (IgAN) and diabetic nephropathy. Studies on CKD (43.75%) and IgAN (37.5%) accounted for the largest proportions.

The CKD sample size was 17,862, with a mean age of 61.93 years old and stage 3–4 in 7 articles. Utilization of the Logistics Regression (LR), Support Vector Machine (SVM) and Random Forest (RF) algorithm accounted for proportions of 15.3%, 10.7% and 9.2%, respectively. The RF algorithm was the most accurate algorithm for predicting CKD prognosis and its AUC was 0.878.

The IgAN sample size was 6127 with a mean age of 34.7 years old in 6 articles. Utilization of the Artificial Neural Network (ANN), LR and Decision Tree (DT) algorithms accounted for proportions of 29.0%, 19.3% and 16.1%, respectively. The ANN algorithm was the most accurate algorithm for predicting IgAN prognosis, with an AUC of 0.933.

### Predictors in the models

Most of the included ML algorithms used demography, comorbidities, and laboratory data from blood and urine samples as predictors, among which, age, sex, hypertension, serum creatinine and 24-h urinary protein were common predictors. However, Dovgan [28] only used comorbidities to construct prediction models. Chen [26] and Schena [30] applied renal biopsy pathology and types of drug therapy as predictors of ML algorithms to establish accurate prediction models. See Additional file 1 for details.

### Outcome indicators

In 12 studies, ESRD was the primary outcome and was defined as such, (1) eGFR < 15 ml/min/1.73 m^2^; (2) the initiation of RRT; (3) renal transplantation. In addition, Xiao et al. [25] used the severity of proteinuria, Feng et al. [23] and Masaki et al. [27] used the progression of CKD stages as outcome indicators for renal disease progression, respectively.

### Makeup of the 6 eligible articles

The 6 eligible articles for data synthesis mainly focus on CKD and IgAN with a total sample size of 12, 534 and a mean age of 42.77 years old. 18 ML algorithms were used to construct progression models with 6 types of predictors mentioned above. Except for Xiao et al. [25], the other 5 researchers took ESRD/RRT as the primary outcomes. Utilization of the ANN, LR, and RF algorithm accounted for proportions of 22.5%, 6.4% and 6.4%, respectively. The optimal prognosis model for predicting IgAN progression was constructed by ANN algorithm. And the optimal prognosis model for predicting CKD progression was constructed by LR algorithm (see Table 1).

### Quality assessment

**Fig. 2:**
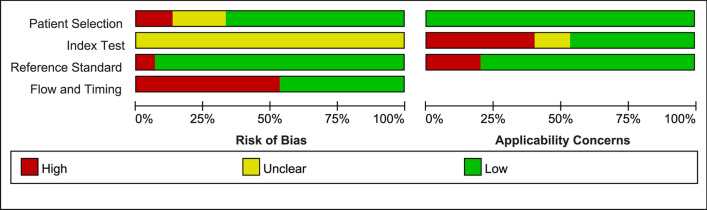
Risk of bias and application concerns graph for the included studies.Red, yellow and green frames correspond to high, unclear and low risk of bias, respectively

**Fig. 3:**
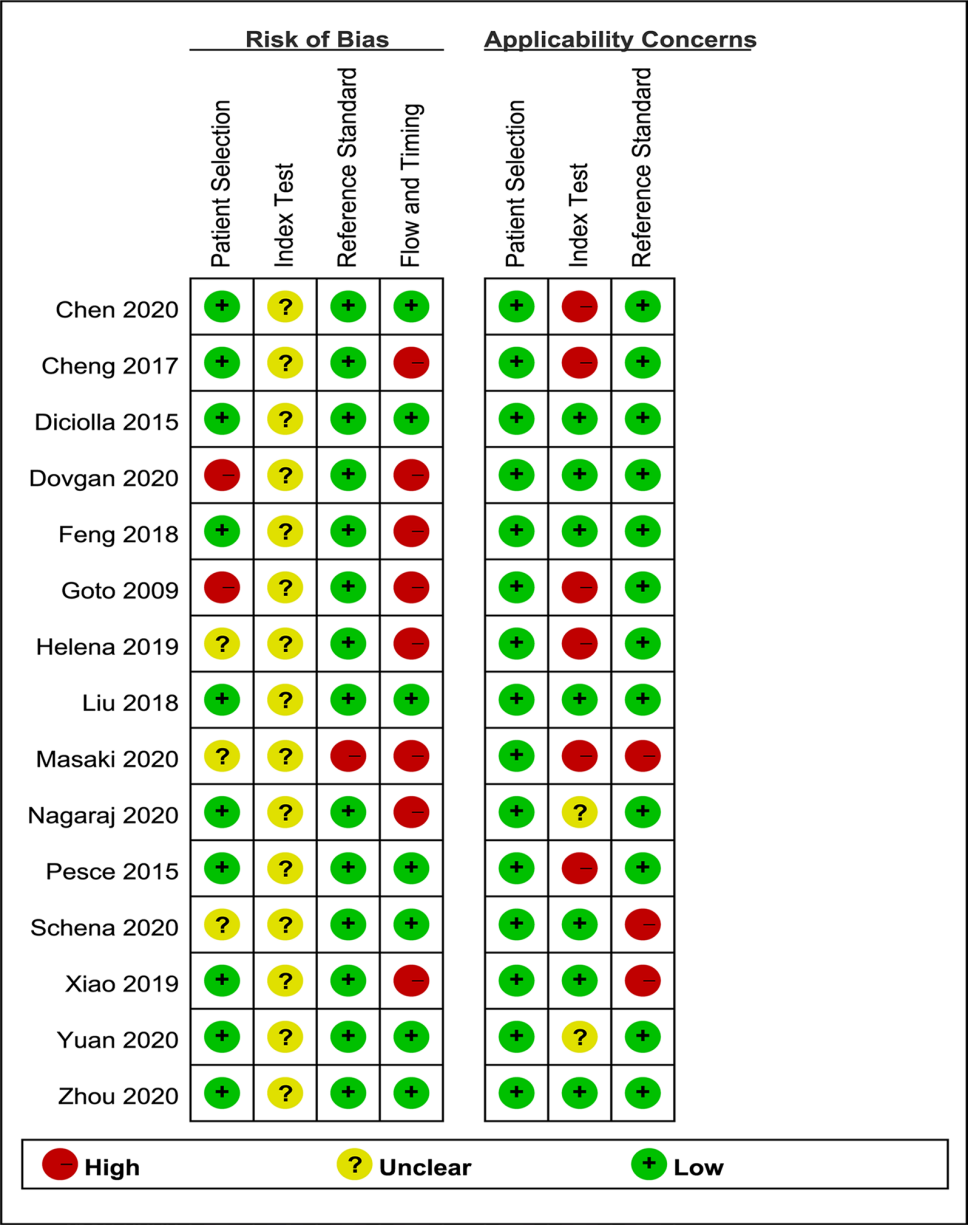
Risk of bias and application concerns summary for the included studies. (+) indicates low risk of bias, (?) indicates unclear risk of bias, (−) indicates high risk of bias

We assessed the studies’ quality with the QUADAS-2 tool. Figure 2 depicts the risk of bias graph, while Fig. 3 presents the risk of bias summary. Bias of the included studies comes primarily from the domains of Index test and Flow and timing. Bias of application concerns pertained primarily to the Index test. Of all the included primary studies, none of the articles were judged as “low risk” on all bias-related domains; 5 (33.3%) were judged as “low concern” on all applicability domains.

## Results of data synthesis

**Fig. 4:**
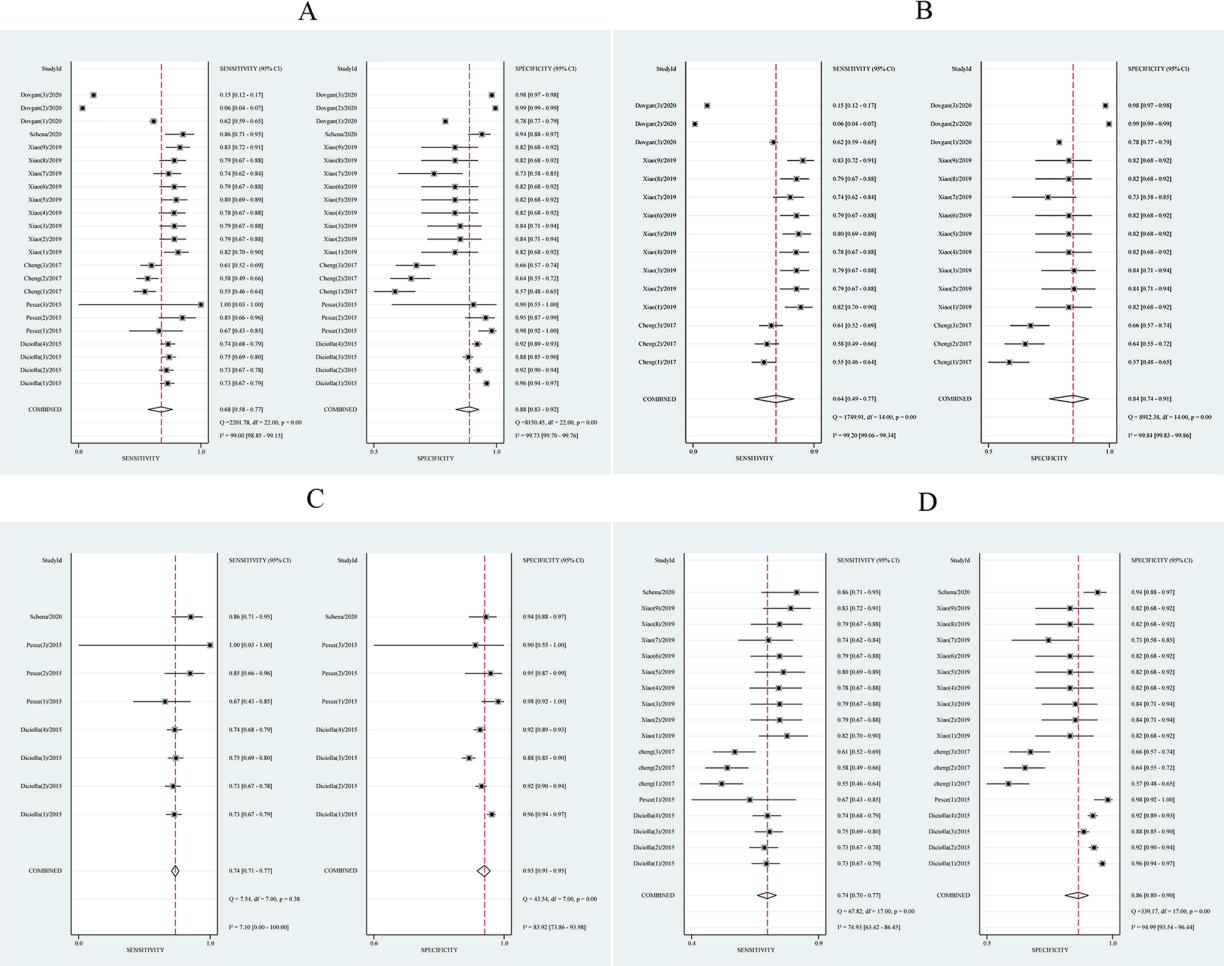
Coupled forest plots for sensitivity and specificity. **A** All single-unit ML algorithms. **B** CKD subgroup. **C** IgAN subgroup. **D** Sensitivity analysis after eliminating outliers and data with small sample sizes. The gray square with a black point in the center showed study specific estimates of sensitivity and specificity. The width of solid black line showed their 95% confidence intervals. The diamond at the bottom of the figure was a combination of single-unit ML algorithm.The center of diamond represented the point estimates, and the width of diamond represented 95% confidence intervals

**Fig. 5:**
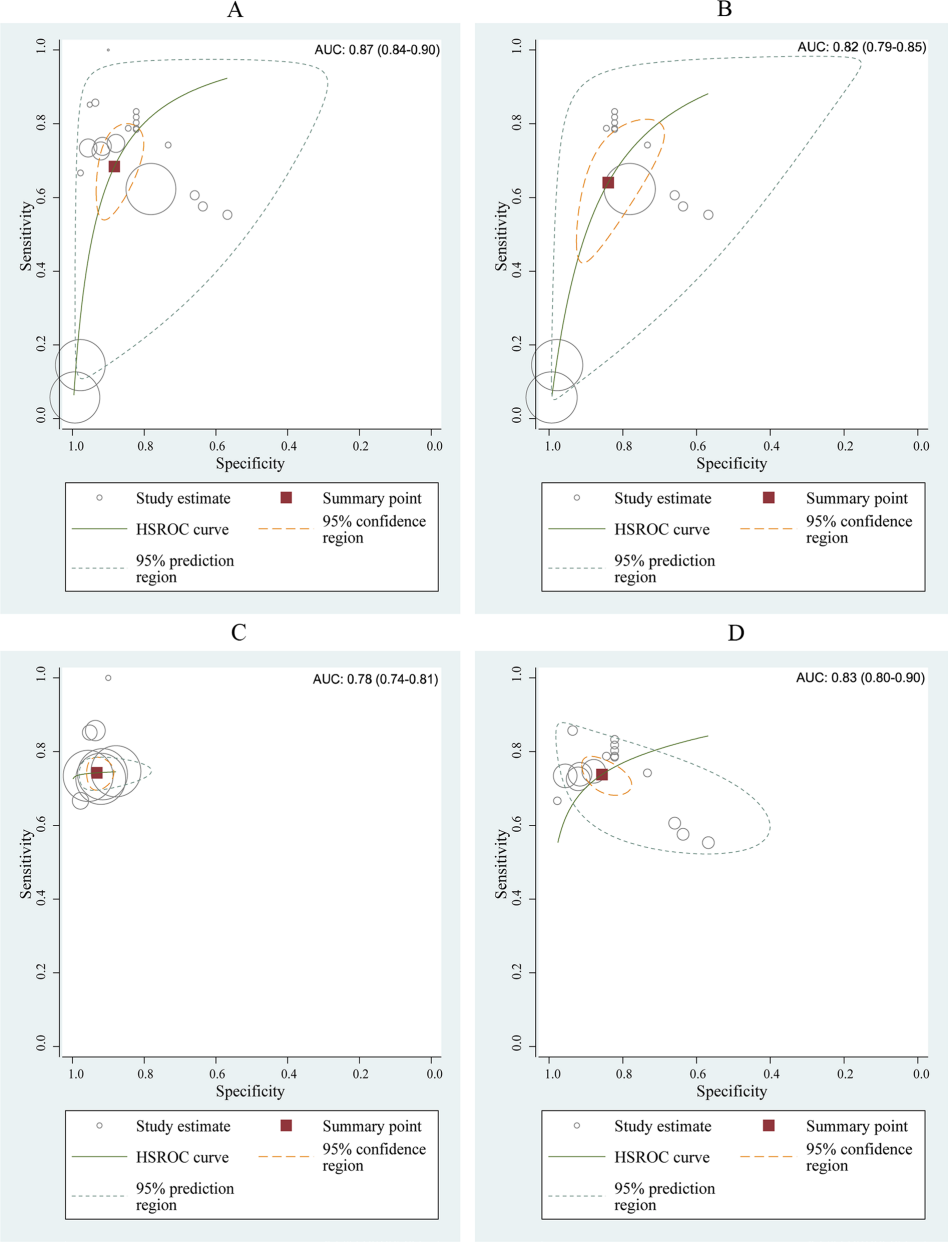
HSROC curve with 95% confidence region and prediction region. **A** All single-unit ML algorithms with AUC of 0.87. **B** CKD subgroup with AUC of 0.82. **C** IgAN subgroup with AUC of 0.78. **D** Sensitivity analysis after eliminating outliers and data with small sample sizes with AUC of 0.83. Each circle represents a single-unit ML algorithm. The curve represents the summary receiver operating characteristic curve for all single-unit ML algorithm. The red square represents the summary estimate of test performance. The zone outlines represent the 95% confidence and 95% prediction regions of the summary estimate, respectively

Extracted data was synthesized using the bivariate model and the HSROC model without accounting for possible covariates explaining heterogeneity. The correlation coefficient was—0.53 and asymmetrical parameter β was 0.015 (*P* > 0.05) which indicated a trade-off between sensitivity and specificity. The ML algorithms exhibited a pool sensitivity of 0.68 (0.58–0.77) and a pool specificity of 0.87 (0.84–0.90) (Fig. 4A). The AUC of the HSROC curve was 0.87 (0.84–0.90) (Fig. 5A), and the DOR was 16.34.

**Fig. 6:**
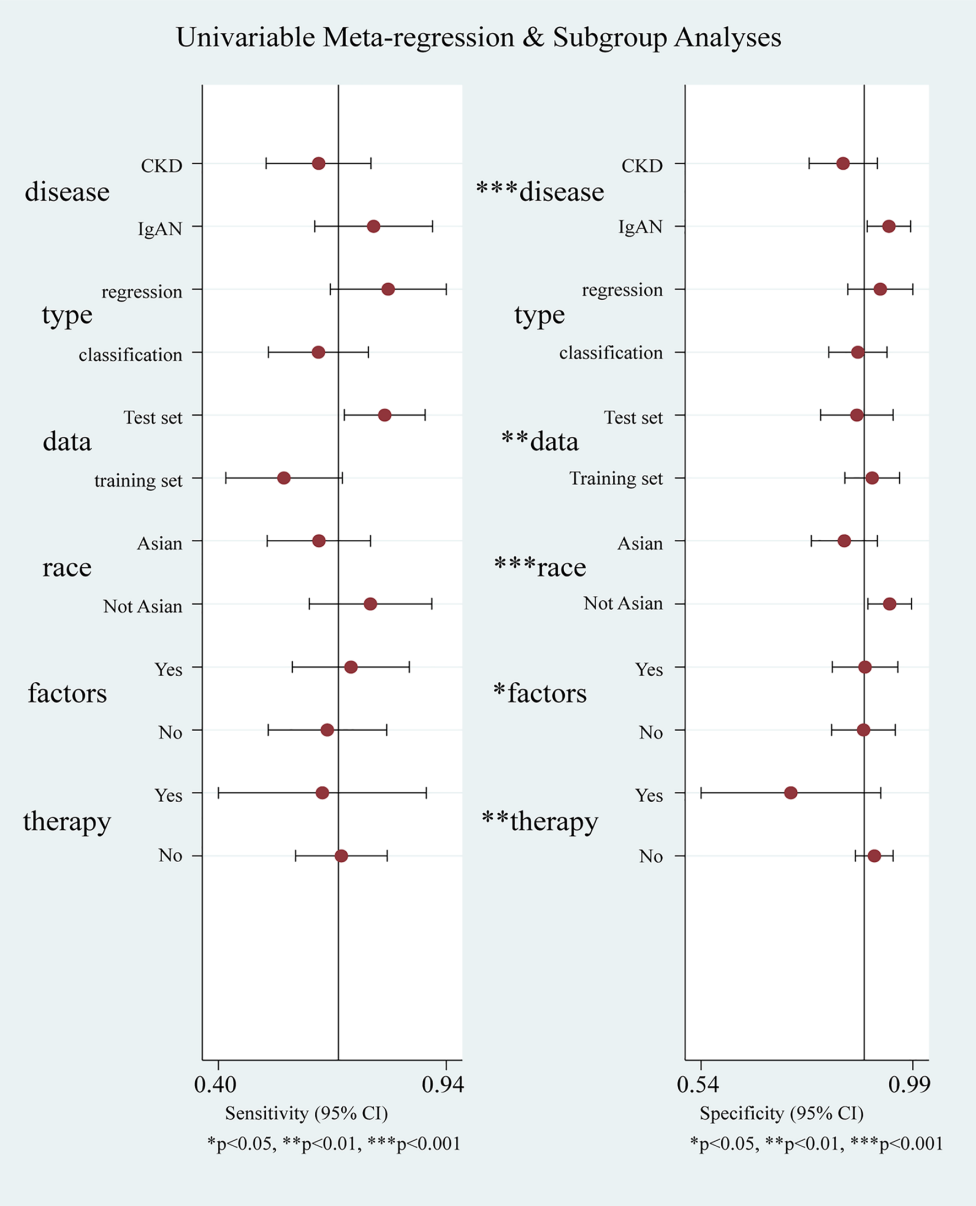
Univariate meta-regression plot of all single-unit ML algorithms. The red point represents the result of the individual combination of the subgroup into which each independent variable is divided. The width of solid black line showed their 95% confidence intervals. “*” means that the effects of independent variables on the pool sensitivity and specificity were statistically significant

The I^2^ for pool sensitivity and specificity were 99.0% and 99.7%, respectively, which indicated the potential heterogeneity. Considering that there were multiple sources of heterogeneity, we conducted a META regression and determined that kidney disease types, algorithm and parameters, dataset, predictors and race were the influential factors (See Fig. 6). After that, we conducted a subgroup analysis.

According to the results of subgroup analysis based on kidney disease types, the ML algorithm’s AUC and DOR for predicting CKD prognosis was 0.82 (0.79–0.85) and 9.31, respectively. The pool sensitivity was 0.64 (0.49–0.77) with an I^2^ of 99.20%, and the pool specificity was 0.84 (0.74–0.91) with an I^2^ of 99.84% (Figs. 4B, 5B). The ML algorithm’s AUC and DOR for predicting IgA nephropathy prognosis was 0.78 (0.74–0.81) and 39.27,respectively. The pool sensitivity was 0.74 (0.71–0.77) with an I^2^ of 7.10%, and the pool specificity was 0.93 (0.91–0.95) with an I^2^ of 83.92% (Figs. 4C, 5C). See Table 2, Additional file 2 and Additional file 3 for more details of subgroup analysis.

**Table 2:** Summary of meta-analysis and subgroup analysis

Subgroup	Number of ML algorithms	Sensitivity (95% CI)	Specificity (95% CI)	AUC (95% CI)	Correlation coefficient	β	DOR
Total DTA	23	0.68 (0.58–0.77)	0.88 (0.83–0.92)	0.87 (0.84–0.90)	− 0.53	0.015	16.34
*Type of KD*							
CKD	15	0.64 (0.49–0.77)	0.84 (0.74–0.91)	0.82 (0.79–0.85)	− 0.77	− 0.036	9.31
IgAN	8	0.74 (0.71–0.77)	0.93 (0.91–0.95)	0.78 (0.74–0.81)	− 1.0	3.781	39.27
*ML algorithm type*							
Classification	16	0.64 (0.50–0.76)	0.87 (0.79–0.92)	0.84 (0.81–0.87)	− 0.66	0.021	11.75
Regression	7	0.80 (0.74–0.84)	0.91 (0.86–0.95)	N/A	1.0	6.044	41.09
*Dataset type*							
Training set	11	0.56 (0.37–0.73)	0.90 (0.80–0.95)	0.83 (0.80–0.86)	− 0.57	0.074	11.40
Testing set	12	0.79 (0.76–0.82)	0.86 (0.81–0.90)	0.81 (0.77–0.84)	− 1.0	3.693	23.33
*Pathology*							
Y	11	0.71 (0.66–0.76)	0.89 (0.80–0.94)	N/A	1	1.086^a^	19.46
N	12	0.65 (0.46–0.81)	0.87 (0.78–0.93)	0.86 (0.83–0.89)	− 0.53	− 0.172	12.92
*Race*							
Asian	16	0.64 (0.49–0.77)	0.84 (0.75–0.91)	0.82 (0.79–0.86)	− 0.76	− 0.042	9.53
Not Asian	7	0.74 (0.71–0.77)	0.93 (0.91–0.95)	0.78 (0.74–0.81)	− 1	3.806	10.95

*DTA* diagnostic test accuracy, *KD* kidney disease, *CKD* chronic kidney disease, *IgAN* Immunoglobulin A Nephropathy, *ML* machine learning, *Y* Yes, *N* No

^a^*P* < 0.01

**Fig. 7:**
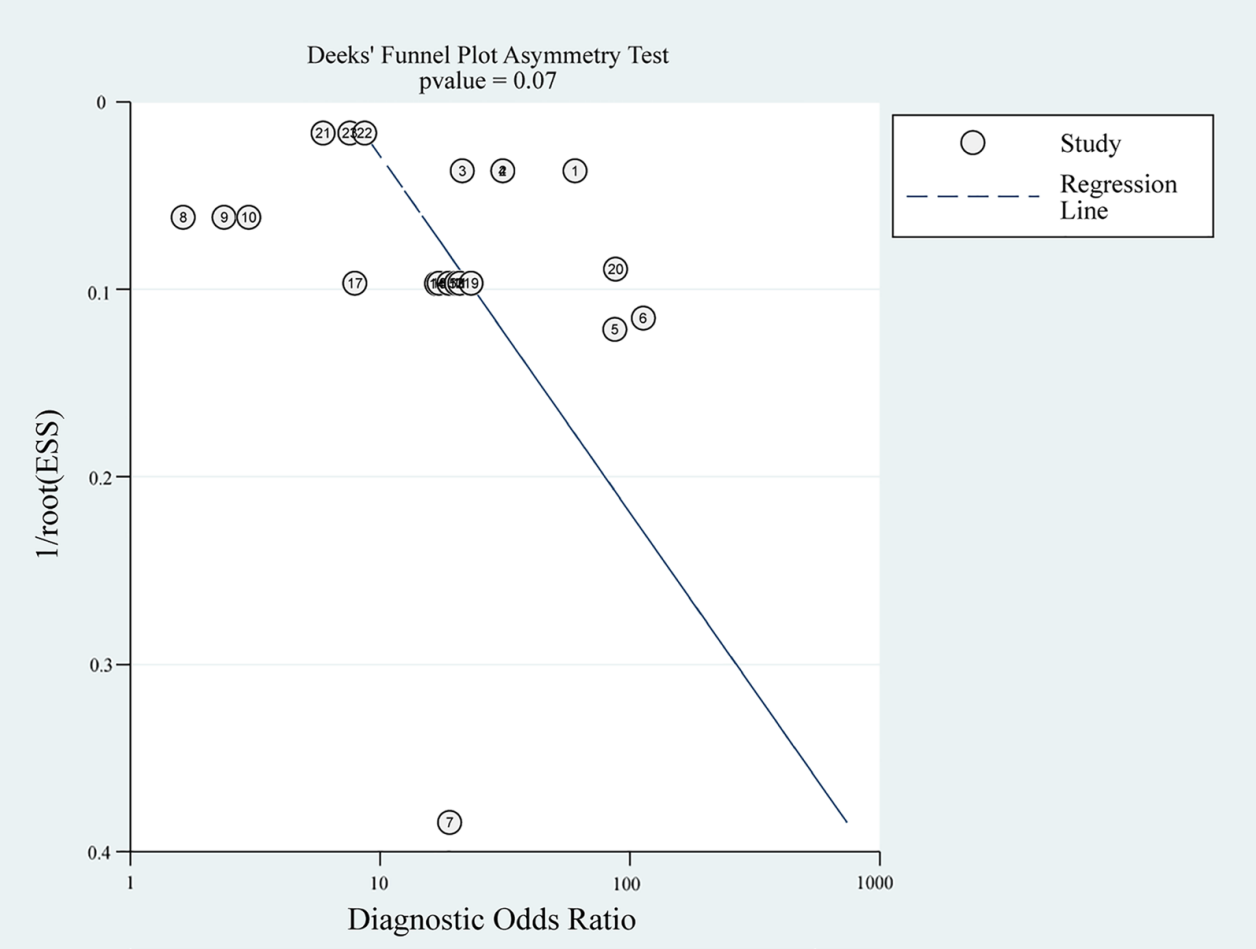
Deek’s funnel plot of all single-unit ML algorithms. Each circle represents a single-unit ML algorithm

We found outliers by observing the forest plot and HSROC curve. We performed sensitivity analysis and reapplied the bivariate and HSROC model after excluding the outliers. Which showed that ML algorithm’s AUC and DOR was 0.83 (0.80–0.90) and 16.80, respectively. The pool sensitivity was 0.74 (0.70–0.77) with an I^2^ of 74.93%, and the pool specificity was 0.86 (0.80–0.90) with an I^2^ of 99.84% (see Figs. 4D, 5D). Additionally, we found that Deek's funnel plot (Fig. 7) was symmetrical, and there was no evidence of publication bias in asymmetric tests (*P* = 0.07).

## Discussion

Our study indicates that ML algorithms did not pool a balance between sensitivity and specificity, which had exceptional accuracy, with an AUC of 0.87, and strong specificity (0.88), but weak sensitivity (0.68) in predicting adverse outcomes, progress to ESRD or initiation of RRT, among both CKD and IgAN patients. This result indicates that recent ML algorithms have low misdiagnosis rates, but significant probability of missed diagnosis, which means that its ability to detect patients with kidney function progression is not strong enough. ML algorithms need optimization because we aim to identify patients at risk. The main reason for the decrease in sensitivity may be the low end-point incidence rate with insufficient follow up time. As shown in the results, only 16.3% of patients reached the end point. In previous studies, a mean follow-up time of at least 5 years has been required to project whether patients with CKD would progress to ESRD [32]. However, in the studies included for data synthesis, the mean follow up times were just 1.5 and 3.0 years, in Cheng’ s and Xiao’ s research, respectively. Sufficient follow-up time is needed to establish prediction models.

The technical superiority of ML algorithm-based prediction models over traditional models is well established. Most previous models have performed well in internal validation, but their capacity for generalization is uncertain because only a portion of the studies have been externally validated. As shown in the subgroup analysis, we found that in the test set group, the pool sensitivity was 0.79 and the pool specificity was 0.86, which indicated an exceptional accuracy in external verification. This is because the goal of machine learning is to fit the models with new samples [33]. Furthermore, in the process of using ML algorithms, it is necessary to first divide the data into a training set and a test set. After learning some potential rules from the training set, the rudimentary model can be verified on the test set. In the included studies, the validation set was used for verification in modeling. However, not all articles reported the results of ML algorithms performed on validation sets, as there is no unified standard for reporting [34]. In order to standardize the research reporting process, and to gain a more comprehensive understanding of ML algorithms, we suggest that future research report the results of both training sets and validation sets. They also show a greater capacity for generalization than traditional statistical methods. However, the small sample size in Pesce’s study’ s test set may have led to high accuracy.

However, when modeling, ML algorithm type should be chosen deliberately. Our study focused on CKD and IgAN, ANN and XGBoost algorithm have been utilized successfully in the field of IgAN [14]. However, regardless of the studies on early stage CKD diagnosis [35–38], there is a lack of studies predicting its outcome. Our study has shown that it is feasible to use ML algorithms to build progression models for CKD. Recent studies have focused on the RF, LR and SVM algorithms which can produce accurate predictions and deserve further study. As for the strengths, the RF and SVM are classification algorithms can produce a qualitative index which can intuitively reflect the occurrence of an outcome event by summarizing and classifying the data characteristics. However, the LR algorithm can utilize as regression algorithm and can predict the probability of an outcome event. But they also have weaknesses. The probability cannot be known when using the classification algorithm, while the regression algorithm-based prediction models cannot produce direct conclusions. Thus, it is necessary to determine the cut-off point.

Additionally, which predictors to use to construct prediction models is undergoing debate. As shown in our subgroup analysis, renal biopsy pathology plays an important role in prognosis predicting. Studies that used pathology had a pool sensitivity of 0.71 (0.66–0.76) and a pool specificity of 0.89 (0.80–0.94). While those without pathology had a pool sensitivity of 0.65 (0.46–0.81) and a pool specificity of 0.87 (0.78–0.93). Which indicate that high quality pathology data optimized the accuracy of prediction models. However, only a small minority of patients can provide pathology data. Because renal biopsy is an invasive manipulation, for which not all patients have indication. Furthermore, there are great differences in renal biopsy specimen preparation and diagnosis for there is no unified or standardized pathological diagnosis mode.

However, there are evidences indicated that CKD prognostic ML prediction models using laboratory data from blood and urine samples are also accurate [22]. Moreover, CKD patients have the most comorbidities [39] correlated with disease prognosis, and there is evidence of comorbidities’ effectiveness for modeling [9, 40]. We believe that with the development of the electronic medical record (EMR) system, [15] the quantity of comorbidity data will grow with its quality improves. Therefore, exploring the use of laboratory data from blood and urine samples and comorbidities as the predictors for modeling.

As for the outcomes, ESRD occurrence and the time to start RRT were the end points which caught the most attention from researchers. Researchers seem to have been less concerned about Major Adverse Cardiovascular Events (MACE) and all-cause mortality. Considering that MACE is the leading cause of death in kidney disease patients [7], we believe that using ML algorithms to predict risk MACE occurrence is also meaningful.

After data synthesis, we found significant heterogeneity between the studies. This may have been due to heterogeneity of kidney diseases, algorithms and parameters, datasets, predictors or race. Thus, we should be cautious when interpreting the results.

The 15 studies in our systematic review have a moderate to severe risk of bias in methodology. This was because we did not have enough information to determine whether the researchers had interpreted the results knowing patients’ outcome. And in some articles, some data was left out of the analysis because the dataset needed to be divided into a training set and a test set (see Additional file 1 for more details).

However, we also found studies with low risk of bias. From these, we found several prediction models with high accuracy which had used common clinical data as predictors. These included Diciolla’ s ANN model whose accuracy was 0.901, Liu’ s RF model whose AUC was 0.9729, Chen’s XGBoost model whose C-Statistic was 0.89 and Yuan’ s RF model whose AUC was 0.878. Based on the results above, we offer several recommendations for clinicians. When predicting whether IgAN patients will progress to ESRD, we recommend either Diciolla’s ANN model or Chen’s XGBoost model, assuming we can obtain patients’ renal biopsy pathology data. However, when this data is unavailable, we recommend Liu’s RF model. When predicting whether CKD patients will progress to ESRD, we suggest Yuan’s RF model. However, note that Yuan’s model is only suitable for CKD stage 3 patients.

## Limitations

1. We found that the sources of heterogeneity were multifaceted, and the high heterogeneity persisted after subgroup analysis. Furthermore, the covariate had a significant influence on the pool specificity. We also believe that ML algorithm type is an important source of heterogeneity. We found that multiple types of ML algorithms were utilized, but few studies focused on one. This makes it impossible to collect enough data to evaluate the performance of a specific type of ML algorithm. Therefore, we cannot eliminate the heterogeneity, and further studies are needed.
2. We utilized data transformation during data extraction. This may have resulted in bias because most of the included studies reported a mean accuracy index without specific TP, FP, FN or TN data.
3. Considering the difference in generalizability, our results might not reflect the actual power of ML algorithms. This is because we synthesized data extracted from the training set and test set, which need improvement in future studies.
4. The combination of multiple ML algorithms is superior to utilizing a single ML algorithm. However, we only synthesized data extracted from a single ML algorithm. This may have caused us to underestimate the accuracy. This is because we cannot get enough data, since few studies have combined more than two ML algorithms during modeling. Furthermore, the type of ML algorithms they utilized for combination varied.
5. The last search was performed on October 31, 2020. After that, we spent almost 10 months screening the retrieved studies, which could affect the timeliness of this study.

## Conclusion

ML algorithms are a tool for unearthing the rules of big data, and prediction models which incorporate them have exceptional accuracy in predicting kidney disease patients’ poor prognosis during clinical practice. The use of ML algorithms can help clinicians detect patients at high risk of kidney function progression in the early stages. In this way, they can receive treatment and management in time. In sum, we suggest the gradual incorporation of ML algorithm-based prediction models into clinical practice.

## Supplementary Information

**Additional file 1: Method S1–S4.** Search Strategies. **Method S5.** Exclusion criteria for articles. **Method S6.** QUADAS-2 coding manual for primary studies included. **Table S1.** QUADAS-2 gradings for each primary study. **Table S2.** Predictors used in each primary study.

**Additional file 2: Figure S1.** HSROC curve for classification algorithm group. **Figure S2.** HSROC curve for regression algorithm group. **Figure S3.** HSROC curve for training set group. **Figure S4.** HSROC curve for test set group.

**Additional file 3: Figure S5.** HSROC curve for subgroup used renal biopsy pathology as a predictor. **Figure S6.** HSROC curve for subgroup did not use renal biopsy pathology as a predictor. **Figure S7.** HSROC curve for Asian group. **Figure S8.** HSROC curve for non-Asian group.

## Data Availability

All data generated or analysed during this study are included in this manuscript [and its supplementary information files], and available from the corresponding author upon reasonable request. Links of database analysed in this manuscript. 1. PubMed, PubMed (http://nih.gov/). 2. EMBASE, Embase. 3. Cochrane Central Register of Controlled Trials, Cochrane|Trusted evidence. Informed decisions. Better health. 4. Chinese Biomedicine Literature Database, https://sinomed.ac.cn/. 5. Chinese National Knowledge Infrastructure, https://cnki.net/. 6. Wanfang Database, https://wanfangdata.com.cn/index.html. 7. VIP Database, https://www.cqvip.com/.

## Abbreviations

ML: Machine learning
HSROC model: The hierarchical summary receiver operating characteristic model
CKD: Chronic kidney disease
IgAN: Immunoglobulin A nephropathy
ESRD: End stage renal diseases
AUC: Area under curve
RRT: Renal replacement therapy
PRISMA: Preferred reporting items for systematic reviews and meta-analyses statement
eGFR: Estimated glomerular filtration rate
MeSH: Medical subject headings
TP: True positive
FP: False positive
TN: True negative
FN: False negative
PPV: Positive predictive value
NPV: Negative predictive value
QUADAS-2: Quality assessment of diagnostic accuracy studies 2
DOR: Diagnostic odds ratios
LR: Logistics regression
SVM: Support vector machine
RF: Random forest
ANN: Artificial neural network
DT: Decision tree
EMR: Electronic medical record
DKD: Diabetes kidney disease
PRO: Proteinuria
NFS: Neural fuzzy systems
ElasticN: Elastic net
KNN: K nearest neighbors
NN: Nearest neighbors
SGD: Stochastic gradient descent
BDTs: Bagging decision trees
CNN: Convolutional neural network
CART: Classification and regression
Lasso: Lasso regression
Ridge: Ridge regression
NNET: Neural network
FNN: Feed-forward neural network
DTA: Diagnostic test accuracy
KD: Kidney disease
