# Supplementary material for: Machine learning algorithms’ accuracy in predicting kidney disease progression: a systematic review and meta-analysis
Source: BMC Med Inform Decis Mak. 2022 Aug 1;22:205. doi: 10.1186/s12911-022-01951-1 (PMC9341041; doi:10.1186/s12911-022-01951-1)
Supplement: Supplementary file 1 — Additional file 1: Method S1–S4. Search Strategies. Method S5. Exclusion criteria for articles. Method S6. QUADAS-2 coding manual for primary studies included. Table S1. QUADAS-2 gradings for each primary study. Table S2. Predictors used in each primary study. [file 12911_2022_1951_MOESM1_ESM.docx]

**Machine Learning Algorithms’ Accuracy in Predicting Kidney Disease Progression:**

**A Systematic Review and Meta-analysis**

**Additional file 1:**

**Method S1. Search Strategies of Embase**

1 'artificial intelligence'/exp

2 'machine learning'/exp

3 'deep learning'/exp

4 ('supervise machine learning' OR 'unsupervised machine learning')/exp

5 'algorithm'/exp

6 'statistical model'/exp

7 'classification'/exp OR 'regression analysis'/exp OR 'cluster analysis'/exp OR 'logistic model'/exp OR 'decision tree'/exp OR 'artificial neural network'/exp OR 'support vector machine'/exp OR 'principal component analysis'/exp

8 1 OR 2 OR 3 OR 4 OR 5 OR 6 OR 7

9 clustering*:ab,ti

10 'training sample':ab,ti OR 'test sample':ab,ti

11 'logistic* regression':ab,ti

12 'linear modelling':ab,ti

13 mp:ab,ti

14 'error backpropagation':ab,ti

15 'genetic algorithm':ab,ti

16 fuzzy*:ab,ti

17 'self-organizing map':ab,ti

18 'deep belief network':ab,ti

19 bayesian:ab,ti

20 'expectation maximization':ab,ti

21 boosting:ab,ti OR adaboost:ab,ti OR lpboost:ab,ti OR xgboost:ab,ti

22 bagging:ab,ti

23 'random forest':ab,ti

24 'k means':ab,ti OR 'k-nearest neighbor':ab,ti OR 'k model':ab,ti

25 'isometric mapping':ab,ti

26 'feature selection':ab,ti

27 lasso:ab,ti

28 markov*:ab,ti

29 'greedy algorithm':ab,ti

30 boltzmann*:ab,ti

31 'convolutional neural network':ab,ti OR convolution:ab,ti

32 'classification tree':ab,ti

33 'regression tree':ab,ti

34 'dimension reduction':ab,ti

35 perceptron*:ab,ti

36 ensemble:ab,ti

37 9 OR 10 OR 11 OR 12 OR 13 OR 14 OR 15 OR 16 OR 17 OR 18 OR 19 OR 20 OR 21 OR 22 OR 23 OR 24 OR 25 OR 26 OR 27 OR 28 OR 29 OR 30 OR 31 OR 32 OR 33 OR 34 OR 35 OR 36

38 'kidney disease'/exp

39 'nephrology'/exp

40 'urinary tract disease'/exp

41 kidney*:ab,ti OR 'kidney disease*':ab,ti OR 'kidney disorder*':ab,ti OR 'renal disease*':ab,ti

42 nephropath*:ab,ti

43 'urologic* disease':ab,ti)

44 38 OR 39 OR 40 OR 41 OR 42 OR 43

45 'disease exacerbation'/exp

46 'pathologic processes'/exp

47 'outcome assessment'/exp

48 'outcome and health care quality'

49 'prognosis'/exp

50 'treatment outcome'/exp

51 45 OR 46 OR 47 OR 48 OR 49 OR 50

52 'disease progression':ab,ti OR prognoses:ab,ti

53 'prognostic factors':ab,ti

54 'patient relevant outcome':ab,ti

55 'clinical effectiveness':ab,ti OR 'treatment efficacy':ab,ti OR 'clinical efficacy':ab,ti

56 52 OR 53 OR 54 OR 55

57 8 AND 37 AND 44 AND 51 AND 56 **154,255**

**Method S2. Search Strategies of Pubmed**

1 artificial intelligence[MeSH Terms]

2 machine learning[MeSH Terms]

3 deep learning[MeSH Terms]

4 (supervise machine learning OR unsupervised machine learning)[MeSH Terms]

5 algorithm[MeSH Terms]

6 statistical model[MeSH Terms]

7 classification[MeSH Terms] OR regression analysis[MeSH Terms] OR cluster analysis[MeSH Terms] OR logistic model[MeSH Terms] OR decision tree[MeSH Terms] OR artificial neural network[MeSH Terms] OR support vector machine[MeSH Terms] OR principal component analysis[MeSH Terms]

8 1 OR 2 OR 3 OR 4 OR 5 OR 6 OR 7

9 clustering*[Title/Abstract]

10 training sample[Title/Abstract]OR test sample[Title/Abstract]

11 logistic* regression[Title/Abstract]

12 linear modelling[Title/Abstract]

13 mp[Title/Abstract]

14 error backpropagation[Title/Abstract]

15 genetic algorithm[Title/Abstract]

16 fuzzy*[Title/Abstract]

17 self-organizing map[Title/Abstract]

18 deep belief network[Title/Abstract]

19 bayesian[Title/Abstract]

20 expectation maximization[Title/Abstract]

21 boosting[Title/Abstract] OR adaboost[Title/Abstract] OR lpboost[Title/Abstract] OR xgboost[Title/Abstract]

22 bagging[Title/Abstract]

23 random forest[Title/Abstract]

24 k means[Title/Abstract] OR k-nearest neighbor[Title/Abstract] OR k model[Title/Abstract]

25 isometric mapping[Title/Abstract]

26 feature selection[Title/Abstract]

27 lasso[Title/Abstract]

28 markov*[Title/Abstract]

29 greedy algorithm[Title/Abstract]

30 boltzmann*[Title/Abstract]

31 convolutional neural network[Title/Abstract]OR convolution[Title/Abstract]

32 classification tree[Title/Abstract]

33 regression tree[Title/Abstract]

34 dimension reduction[Title/Abstract]

35 perceptron*[Title/Abstract]

36 ensemble[Title/Abstract]

37 9 OR 10 OR 11 OR 12 OR 13 OR 14 OR 15 OR 16 OR 17 OR 18 OR 19 OR 20 OR 21 OR 22 OR 23 OR 24 OR 25 OR 26 OR 27 OR 28 OR 29 OR 30 OR 31 OR 32 OR 33 OR 34 OR 35 OR 36

38 kidney disease[MeSH Terms]

39 nephrology[MeSH Terms]

40 urinary tract disease[MeSH Terms]

41 kidney*[Title/Abstract] OR kidney disease*[Title/Abstract] OR kidney disorder*[Title/Abstract] OR renal disease*[Title/Abstract]

42 nephropath*[Title/Abstract]

43 urologic* disease[Title/Abstract]

44 38 OR 39 OR 40 OR 41 OR 42 OR 43

45 disease exacerbation[MeSH Terms]

46 pathologic processes[MeSH Terms]

47 outcome assessment[MeSH Terms]

48 outcome and health care quality[MeSH Terms]

49 prognosis[MeSH Terms]

50 treatment outcome[MeSH Terms]

51 45 OR 46 OR 47 OR 48 OR 49 OR 50

52 disease progression[Title/Abstract]OR prognoses[Title/Abstract]

53 prognostic factors[Title/Abstract]

54 patient relevant outcome[Title/Abstract]

55 clinical effectiveness[Title/Abstract] OR treatment efficacy[Title/Abstract] OR clinical efficacy[Title/Abstract]

56 52 OR 53 OR 54 OR 55

57 8 AND 37 AND 44 AND 51 AND 56 **21,177**

**Method S3. Search Strategies of Cochrane**

#1 (Artificial Intelligence):ti,ab,kw OR (Machine Learning):ti,ab,kw OR (Deep Learning):ti,ab,kw OR (Algorithms):ti,ab,kw OR (Classification):ti,ab,kw

#2 (Regression Analysis):ti,ab,kw OR (Cluster Analysis):ti,ab,kw OR (Supervise Machine Learning):ti,ab,kw OR (Unsupervised Machine Learning):ti,ab,kw OR (Linear Model):ti,ab,kw

#3 (Logistic Model):ti,ab,kw OR (Decision Trees):ti,ab,kw OR (Neural Networks, Computer):ti,ab,kw OR (Support Vector Machine):ti,ab,kw OR (Principal Component Analysis):ti,ab,kw

#4 #1or#2or#3

#5 (Clustering*):ti,ab,kw OR (training sample):ti,ab,kw OR (test sample):ti,ab,kw OR (Logistic* regression):ti,ab,kw OR (linear modelling):ti,ab,kw

#6 (MP):ti,ab,kw OR (error BackPropagation):ti,ab,kw OR (genetic algorithm):ti,ab,kw OR (Fuzzy*):ti,ab,kw OR (self-organizing Map):ti,ab,kw

#7 (deep belief network):ti,ab,kw OR (Bayesian*):ti,ab,kw OR (expectation maximization):ti,ab,kw OR (Boosting):ti,ab,kw OR (AdaBoost):ti,ab,kw

#8 (LPBoost):ti,ab,kw OR (xgBoost):ti,ab,kw OR (Bagging):ti,ab,kw OR (random forest):ti,ab,kw OR (k-means):ti,ab,kw

#9 (k-nearest neighbor):ti,ab,kw OR (k-model):ti,ab,kw OR (isometric mapping):ti,ab,kw OR (feature selection):ti,ab,kw OR (LASSO):ti,ab,kw

#10 (Markov*):ti,ab,kw OR (greedy algorithm):ti,ab,kw OR (Boltzmann*):ti,ab,kw OR (convolutional neural network):ti,ab,kw OR (classification tree):ti,ab,kw

#11 (regression tree):ti,ab,kw OR (dimension reduction):ti,ab,kw OR (perceptron*):ti,ab,kw OR (ensemble):ti,ab,kw

#12 #5or#6or#7or#8or#9or#10or#11 57761

#13 #4or#12

#14 (Kidney Disease):ti,ab,kw OR (Kidney*):ti,ab,kw OR (kidney disease*):ti,ab,kw OR (kidney disorder*):ti,ab,kw OR (renal disease*):ti,ab,kw 60162

#15 (Nephrology):ti,ab,kw OR (Urologic Diseases):ti,ab,kw OR (nephropath*):ti,ab,kw OR (Urologic* Disease):ti,ab,kw 11709

#16 #14or#15

#17 (Disease Progression):ti,ab,kw OR (Pathologic Processes):ti,ab,kw OR (Patient Outcome Assessment):ti,ab,kw OR (Outcome and Process Assessment, Health Care):ti,ab,kw OR (Prognosis):ti,ab,kw

#18 (Treatment Outcome):ti,ab,kw OR (Disease Exacerbation):ti,ab,kw OR (Prognoses):ti,ab,kw OR (Prognostic Factors):ti,ab,kw OR (Patient Relevant Outcome):ti,ab,kw 348460

#19 (Clinical Effectiveness):ti,ab,kw OR (Treatment Efficacy):ti,ab,kw OR (Clinical Efficacy):ti,ab,kw

#20 #17or#18or#19

#21 #13and#16and#20 **4390**

**Method S4. Search Strategies of Chinese National Knowledge Infrastructure**

(TI=肾 OR TI=泌尿) AND (TKA=人工智能OR TKA=机器学习OR TKA=深度学习 OR TKA=算法OR TKA=分类OR TKA=回归 OR TKA=聚类 OR TKA=监督学习 OR TKA=无监督学习 OR TKA=线性模型 OR TKA= Logistic回归OR TKA=逻辑回归 OR TKA=决策树 OR TKA=感知机 OR TKA=神经网络 OR TKA=基因算法 OR TKA=深度信念网络 OR TKA=贝叶斯 OR TKA= EM算法 OR TKA=随机森林 OR TKA=特征选择 OR TKA=马尔可夫 OR TKA=贪心算法 OR TKA=深度神经网络 OR TKA=玻尔兹曼 OR TKA=卷积神经网络OR TKA=k-means聚类 OR TKA=k临近 OR TKA=集成学习 OR TKA= Boosting OR TKA= Bagging OR TKA=训练集 OR TKA=测试集)

**Method S5. Exclusion criteria for articles.**

1. Patients with urinary calculi, renal cancer, ESRD and those undergoing maintain RRT
2. Models constructed by traditional mathematical methods
3. Reviews, editorials, and letters

**Method S6. QUADAS-2 coding manual for primary studies included.**

**Overall risk of bias:**

If the answers to all signaling questions for a domain are “yes”,then risk of bias can be judged low.If any signaling question is answered “no”,potential for bias exists.The “unclear” category would be used only when insufficient data are reported to permit a judgment.

**Domain 1:Patient Selection**

**Risk of bias：**

1. **Could the selection of patients have introduced bias?**

**Signalling question 1:Was a consecutive or random sample of patients enrolled?**

Code as “Yes” if a consecutive or random sample of participants were recruited for the study and had a time scale during recruitment.Code as “No” if there were no introductions on how to obtain sample of participants.Code as “Unclear” if there was only a description about time scale for recruitment but without a clear indication of the process that how participants were recruited.

**Signalling question 2:Was a case–control design avoided?**

Code as “Yes” if the study did not employ a case-control design,which means that participants were divided into different groups based on their occurrence of outcome events after a period of follow-up time.Code as “No” if the study used a case-control design,which means that participants were grouped based on the occurrence of primary outcomes at the very beginning of the study.

**Signalling question 3:Did the study avoid inappropriate exclusions?**

Inappropriate exclusions refer to situations where an important part of the study participants were excluded from the study based on characteristics that could be related to the results,including demographic information,economic conditions,severity of diseases and comorbidities,etc.Code as “Yes” if the study does not inappropriately exclude participants.Code as “No” if the study inappropriately excludes participants.Code as “Unclear” if there are no enough information to judge weather the study inappropriately excludes participants or not.

**Concerns about applicability：**

**Are there concerns that the included patients do not match the review question?**

Code as “Low” if the study included participants with normal eGFR or CKD stage1-4 patients.Code as “High” if the study included CKD stage5 patients.

**Domain 2:Index test**

**Risk of bias：**

1. **Could the conduct or interpretation of the index test have introduced bias?**

**Signalling question 1:Were** **the index test results interpreted without knowledge of the results of the reference standard?**

Code this item as “Unclear” for all studies.Because we failed to judge whether the researchers interpreted the results of index test without knowledge of the participants’ outcomes.Though retrospective data were used and the exact number of participants who met the outcomes were determined,there are still no enough information to determine whether the researchers knew the outcome of each participant when conducting statistical analysis.

**Signalling question 2:If a threshold was used, was it prespecified?**

Code this item as “Unclear” for all studies.Because none of the articles we included described the use of thresholds.

**Concerns about applicability：**

**Are there concerns that the index test,its conduct,or its interpretation differ from the review question?**

Code as “Low” if the study described the predictors,data processing,parameters and operation process of ML algorithm in detail.Code as “High” if more than one factor described above was missing in the study.Code as “Unclear” if the study only introduced the method of implementing the ML algorithm by software.

**Domain 3:Reference Standard**

**Risk of bias：**

1. **Could the reference standard,its conduct, or its interpretation have introduced bias?**

**Signalling question 1:Is the reference standard likely to correctly classify the target condition?**

Code as “Yes” if the indicators used to evaluate the outcomes in the study can reflect the severity and poor prognosis of nephropathy are objective and have been unanimously recognized by the academic community.Such as the change in Scr,Pro and eGFR or occurrence of ESRD and initiation of RRT,etc.Code as “No” if subjective indicators or the progression of stage were used to evaluate the outcomes.

**Signalling question 2:Were the reference standard results interpreted without knowledge of the results of the index test?**

Code as “Yes” if the researcher was blinded to the participant’s outcomes when diagnosing by ML algorithm based model.Code as “No” if the researcher was not blinded to the participant’s outcomes when diagnosing.Code as “Unclear” if we cannot ascertain whether blinding occurred.

**Concerns about applicability：**

**Are there concerns that the target condition as defined by the** **reference standard does not match the review question?**

Code as “Low” if the reference standard was easy to operate and can determine the deterioration of kidney diseases accurately.Code as “High” if the reference standard was hard to operate and cannot determine the deterioration of kidney diseases.

**Domain 4:Flow and timing**

**Risk of bias：**

**Signalling question 1:Was there** **an appropriate interval between index tests and reference standard?**

Code this item as “Yes” for all studies.Because all studies included had a clear time for the date of completion and we take the patients’ status at the end of the study as the reference,so we can assume that the condition of patients would not change after that.And all of the index test was used after the study period,so it can be deemed that an appropriate interval between index test and reference standard.

**Signalling question 2:Did all patients receive a reference standard?Did all patients receive the same reference standard?**

Code as “Yes” if the reference standard was the occurrence of ESRD and all included participants received it.Code as “No” if the reference standard that all participants received was defined as other indicators.

**Signalling question 3:Were all patients included in the analysis?**

Code as “Yes” if all participants were included in the statistical analysis on both training and test set.Code as “No” if a portion of participants was omitted in the process of statistical analysis.Including missing data cause by the loss of follow up or data matching.And the process of statistical analysis was conducted after the training set and test set were divided.Code as “Unclear” if the article did not describe in detail.

**Table S1. QUADAS-2 gradings for each primary study**

|  | **Patient selection** | | | | | **Index test** | | | | **Reference standard** | | | | **Flow and timing** | | | |
| --- | --- | --- | --- | --- | --- | --- | --- | --- | --- | --- | --- | --- | --- | --- | --- | --- | --- |
|  | S1 | S2 | S3 | Risk of bias | applicability | S1 | S2 | Risk of bias | applicability | S1 | S2 | Risk of bias | applicability | S1 | S2 | S3 | Risk of bias |
| Goto 2009 | Yes | No | Yes | High | Low | U/C | U/C | U/C | High | Yes | Yes | Low | Low | Yes | No | Yes | High |
| Diciolla 2015 | Yes | Yes | Yes | Low | Low | U/C | U/C | U/C | Low | Yes | Yes | Low | Low | Yes | Yes | Yes | Low |
| Pesce 2015 | Yes | Yes | Yes | Low | Low | U/C | U/C | U/C | High | Yes | Yes | Low | Low | Yes | Yes | Yes | Low |
| Cheng 2017 | Yes | Yes | Yes | Low | Low | U/C | U/C | U/C | High | Yes | Yes | Low | Low | Yes | Yes | No | High |
| Feng 2018 | Yes | Yes | Yes | Low | Low | U/C | U/C | U/C | Low | Yes | Yes | Low | Low | Yes | No | No | High |
| Liu 2018 | Yes | Yes | Yes | Low | Low | U/C | U/C | U/C | Low | Yes | Yes | Low | Low | Yes | Yes | Yes | Low |
| Helena 2019 | U/C | Yes | Yes | U/C | Low | U/C | U/C | U/C | High | Yes | Yes | Low | Low | Yes | Yes | No | High |
| Xiao 2019 | Yes | Yes | Yes | Low | Low | U/C | U/C | U/C | Low | Yes | Yes | Low | High | Yes | No | Yes | High |
| Chen 2020 | Yes | Yes | Yes | Low | Low | U/C | U/C | U/C | High | Yes | Yes | Low | Low | Yes | Yes | Yes | Low |
| Dovgan 2020 | Yes | No | Yes | High | Low | U/C | U/C | U/C | Low | Yes | Yes | Low | Low | Yes | No | U/C | High |
| Masaki 2020 | U/C | Yes | Yes | U/C | Low | U/C | U/C | U/C | High | NO | Yes | High | High | Yes | No | Yes | High |
| Nagaraj 2020 | Yes | Yes | Yes | Low | Low | U/C | U/C | U/C | U/C | Yes | Yes | Low | Low | Yes | Yes | No | High |
| Schena 2020 | U/C | Yes | Yes | U/C | Low | U/C | U/C | U/C | Low | Yes | Yes | Low | High | Yes | Yes | Yes | Low |
| Yuan 2020 | Yes | Yes | Yes | Low | Low | U/C | U/C | U/C | U/C | Yes | Yes | Low | Low | Yes | Yes | Yes | Low |
| Zhou 2020 | Yes | Yes | Yes | Low | Low | U/C | U/C | U/C | Low | Yes | Yes | Low | Low | Yes | Yes | Yes | Low |

**Table S2. Predictors used in each primary study**

|  | Demographic | Comorbidities | Blood sample | Urine sample | Pathology | Therapy |
| --- | --- | --- | --- | --- | --- | --- |
| Goto 2009 | DBP | HG | Serum TP、hypoalbuminaemia | PRO,BLD | N/A | N/A |
| Diciolla 2015 | age、gender | hypertension,HG | SCR | 24hPRO | N/A | N/A |
| Pesce 2015 | age、gender | hypertension,HG | SCR | 24hPRO | N/A | N/A |
| Cheng 2017 | age,gender,smoke,alcohol  BQC,exercise,height,weight  waist,hips,SBP,DBP | hypertension,anemia,gout  DM,CVD,HC | SCR、BUN、HB、Hct、WBC、RBC、Ca、P、Na、K、Cl、Mg、SUA、TC、TG、ALB、iPTH、Total Protein、Sugar [AC]、HDL-C-、LDL-C、Fe、Ferritin、Ca × P | PRO、 ACR | N/A | Chinese herbs、Analgesics |
| Feng 2018 | age,gender,BMI,SBP,DBP | N/A | SCR、ALB、Hb、BUN、SUA、Ca、P、Na、K、Mg、Cl、CO2、Glc、CRP | N/A | N/A | N/A |
| Liu 2018 | N/A | hypertension,MAP,HG,DC | eGFR、SCR、BUN、SUA、Hb、ALB、TG、IgM | 24hPRO | T score、Tubular atrophy Interstitial fibrosis、Pathological type、Glomerularsclerosis、C3 staining、Segmentalsclerosis | N/A |
| Helena 2019 | age,gender | N/A | eGFR | ACR | N/A | N/A |
| Xiao 2019 | age,gender,height,weight,BMI | N/A | CRP、ALB、TC、TG、BG、BUN、EGFR、Scr、SUA、SK、Sna、LDL、HDL | 24hPRO | N/A | N/A |
| Chen 2020 | age | hypertension | ALB、SUA、Microscopic hematuria、SCR | PRO | Tubular atrophy interstitial fibrosis、Global sclerosis、Mean mesangial score | N/A |
| Dovgan 2020 | N/A | AGN,CGN,DM,hypertension,HL,PKD,RS,SLE, CHF,CAD,  cerebrovascular disease | N/A | PRO | N/A | N/A |
| Nagaraj 2020 | age,gender,BMI,smoking,DBP,SBP | CVD | SCR、K、HbA1c、ALB、Ca、P、SUA、HDL、LDL | ACR | N/A | N/A |
| Masaki 2020 | N/A | Medical history^1^ | eGFR | PRO | N/A | N/A |
| Schena 2020 | age,gender | hypertension | SCR | PRO | MESCT | RASBs、Corticosteroids/Is |
| Yuan 2020 | age | DM | ALB、TP、TBIL、DBIL、A/G、HGB、Ca、eGFR、HCT、TC、ALT、HDL、SCR、UREA、TC、EPO、MCH、EON、Cl | PRO,SG | N/A | N/A |
| Zhou 2020 | Age,gender,BMI | GN,OrganTx,DM,HTN,Obesity,SA,BMI Spine,Nerve,Hips, Eyes, and Skin,Anemia GI,Thyroid,Psychiatric,Gyn,Asthma Family Hx,DNR,Multiple Myeloma,Sepsis  Hematologic,Cirrhosis | N/A | BLD | N/A | N/A |
| Abbreviation：  **BQC**,Betel quid chewing;**BMI,**body mass index;**SBP,**systolic blood pressure;**DBP,**diastolic blood pressure;**HG**,Histological grade;**DM,**diabetes mellitus;**CVD,**Cardiovascular disease;**HC,**High Cholesterol;**HL,**hyperlipidemia;**MAP,**mean arterial pressure;  **DC,**Disease course;**GN,**glomerulonephritis;**AGN,**acute glomerulonephritis;**CGN,**chronic glomerulonephritis;**PKD,**polycystic kidney disease;**RS,**renal stone;**SLE,**Systemic Lupus Erythematosus,**CHF,**congestive heart failure;**CAD,**coronary artery disease;**OrganTx,**solid organ transplant;**HTN,**Hypertensive heart and chronic kidney disease and malignant hypertension;**SA,**Sleep Apnea;**Spine,**Spondy loarthropathies;**Nerve,**neuropathies;**Hips, Eyes, and Skin,**Hip arthropathies,ophthalmologic,dermatologic;**GI,**Upper and lower gastrointestinal (GI) tract diagnoses;**Family Hx,**tubal ligation family history;**DNR,**do not resuscitate status;  **PRO,**urine protein;**BLD,**haematuria;**SG,**urine specific gravity;**ACR,**Albumin to Creatinine Ratio;  ^1^Not in details | | | | | | |
