# Supplementary material for: Machine learning algorithms’ accuracy in predicting kidney disease progression: a systematic review and meta-analysis
Source: BMC Med Inform Decis Mak. 2022 Aug 1;22:205. doi: 10.1186/s12911-022-01951-1 (PMC9341041; doi:10.1186/s12911-022-01951-1)

**Machine Learning Algorithms’ Accuracy in Predicting Kidney Disease Progression:**

**A Systematic Review and Meta-analysis**

**Additional file 2:**

**Figure S1. HSROC curve for classification algorithm group with AUC of 0.84.**

**
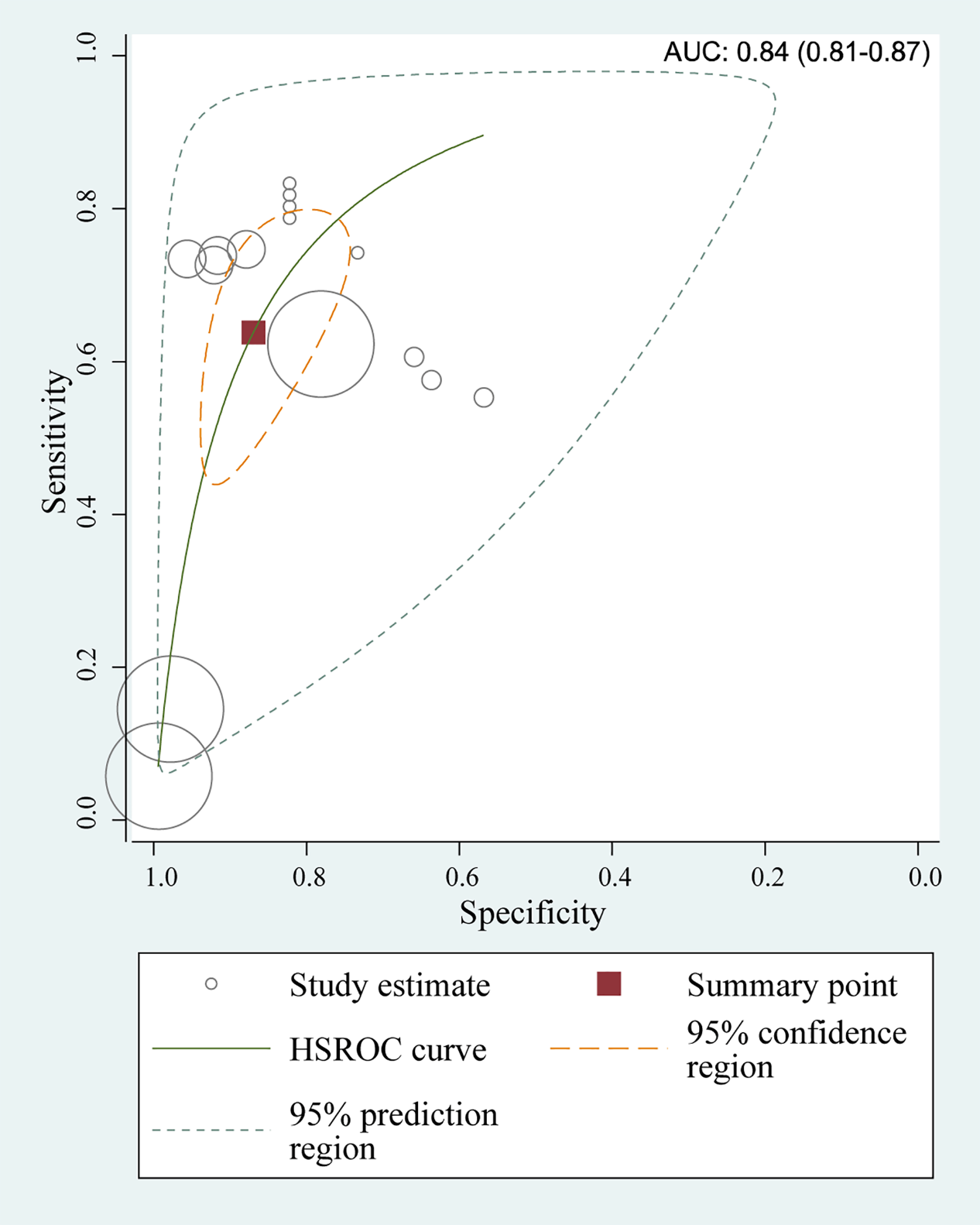
**

**Figure S2. HSROC curve for regression algorithm group.**

**
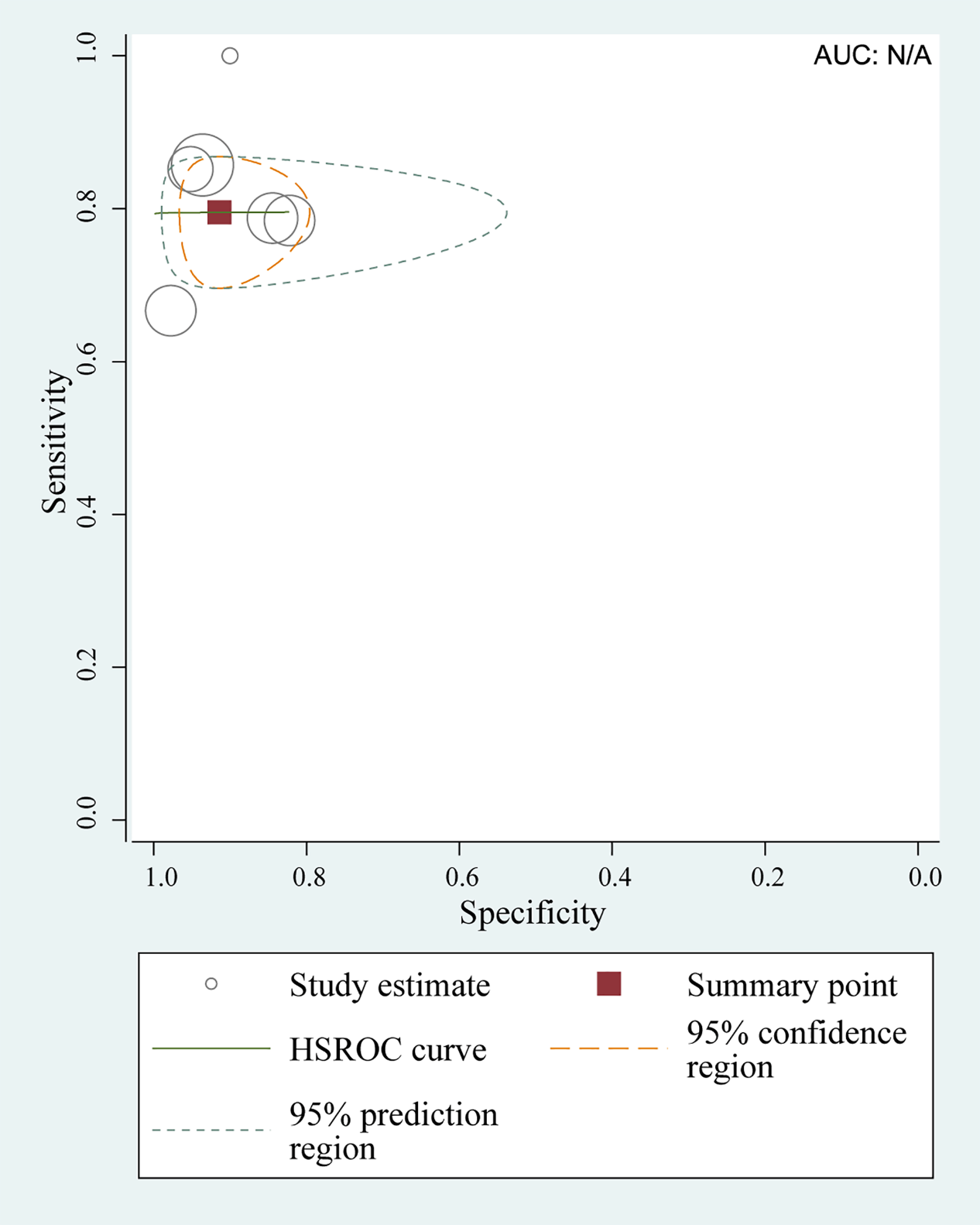
**

**Figure S3. HSROC curve for training set group with AUC of 0.83.**


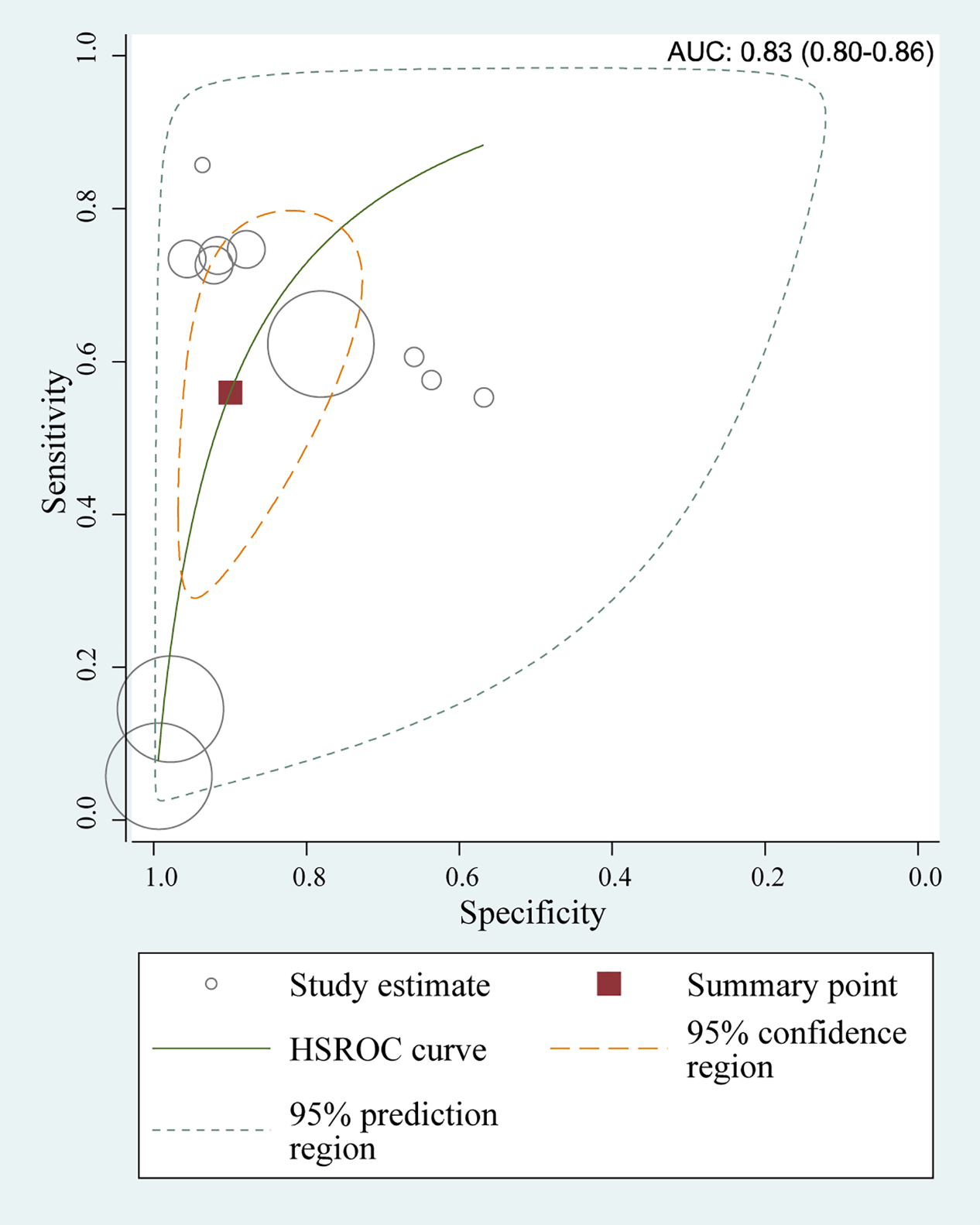


**Figure S4. HSROC curve for test set group with AUC of 0.81.**


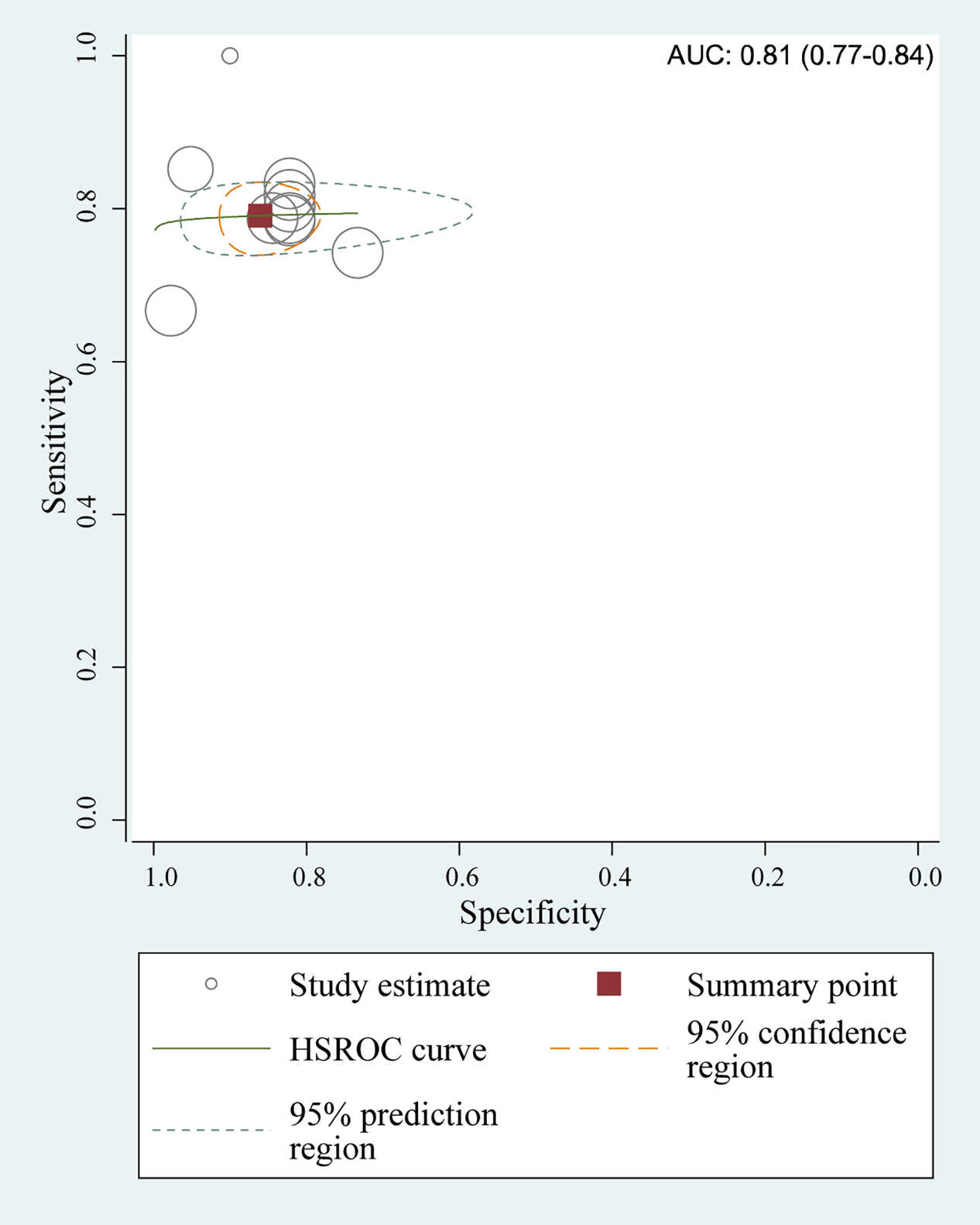

Supplement: Supplementary file 2 — Additional file 2: Figure S1. HSROC curve for classification algorithm group. Figure S2. HSROC curve for regression algorithm group. Figure S3. HSROC curve for training set group. Figure S4. HSROC curve for test set group. [file 12911_2022_1951_MOESM2_ESM.docx]
