# Supplementary material for: Machine learning algorithms’ accuracy in predicting kidney disease progression: a systematic review and meta-analysis
Source: BMC Med Inform Decis Mak. 2022 Aug 1;22:205. doi: 10.1186/s12911-022-01951-1 (PMC9341041; doi:10.1186/s12911-022-01951-1)

**Machine Learning Algorithms’ Accuracy in Predicting Kidney Disease Progression:**

**A Systematic Review and Meta-analysis**

**Additional file 2:**

**Figure S5. HSROC curve for subgroup used renal biopsy pathology as a predictor.**


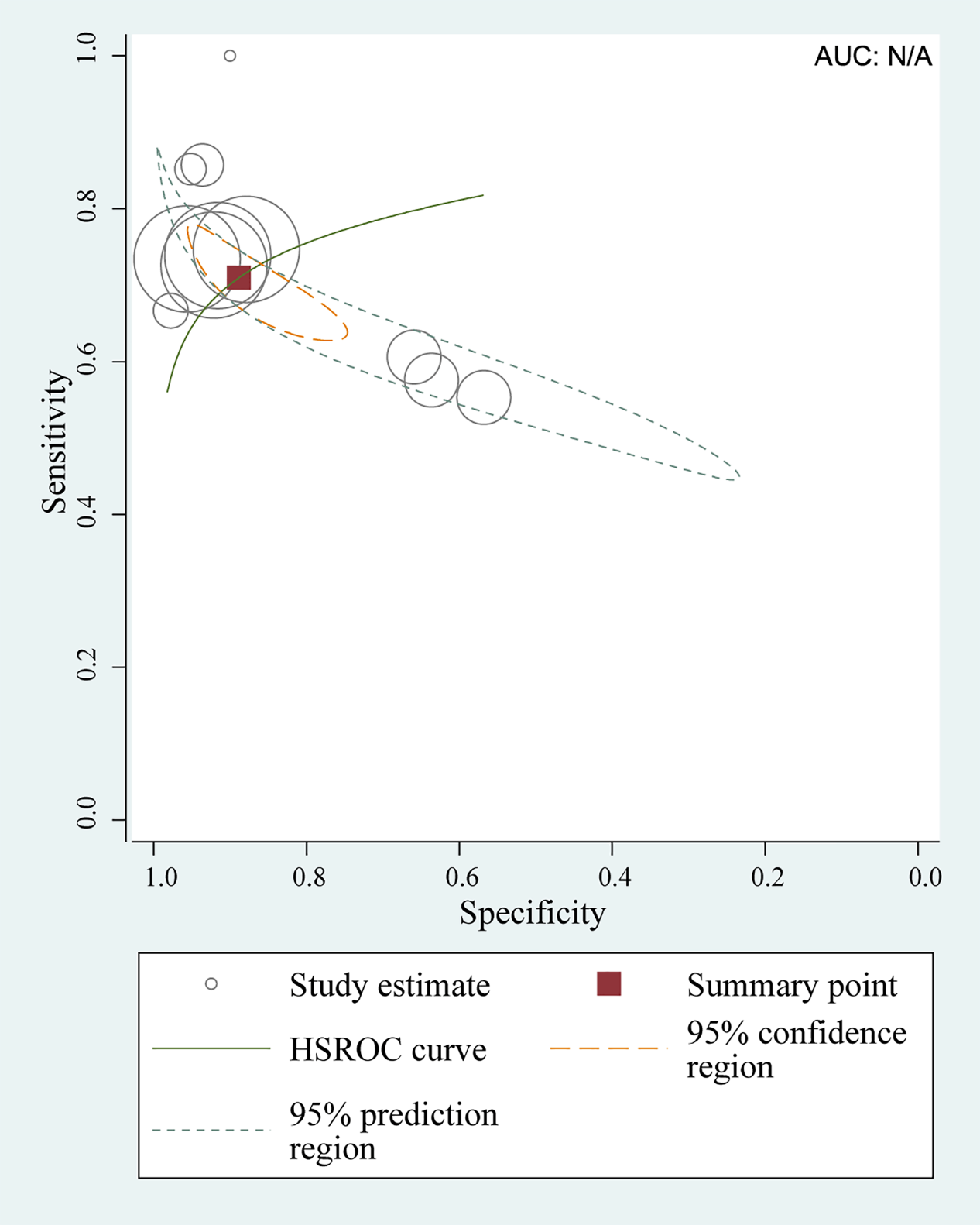


**Figure S6. HSROC curve for subgroup did not use renal biopsy pathology as a predictor with AUC of 0.86.**


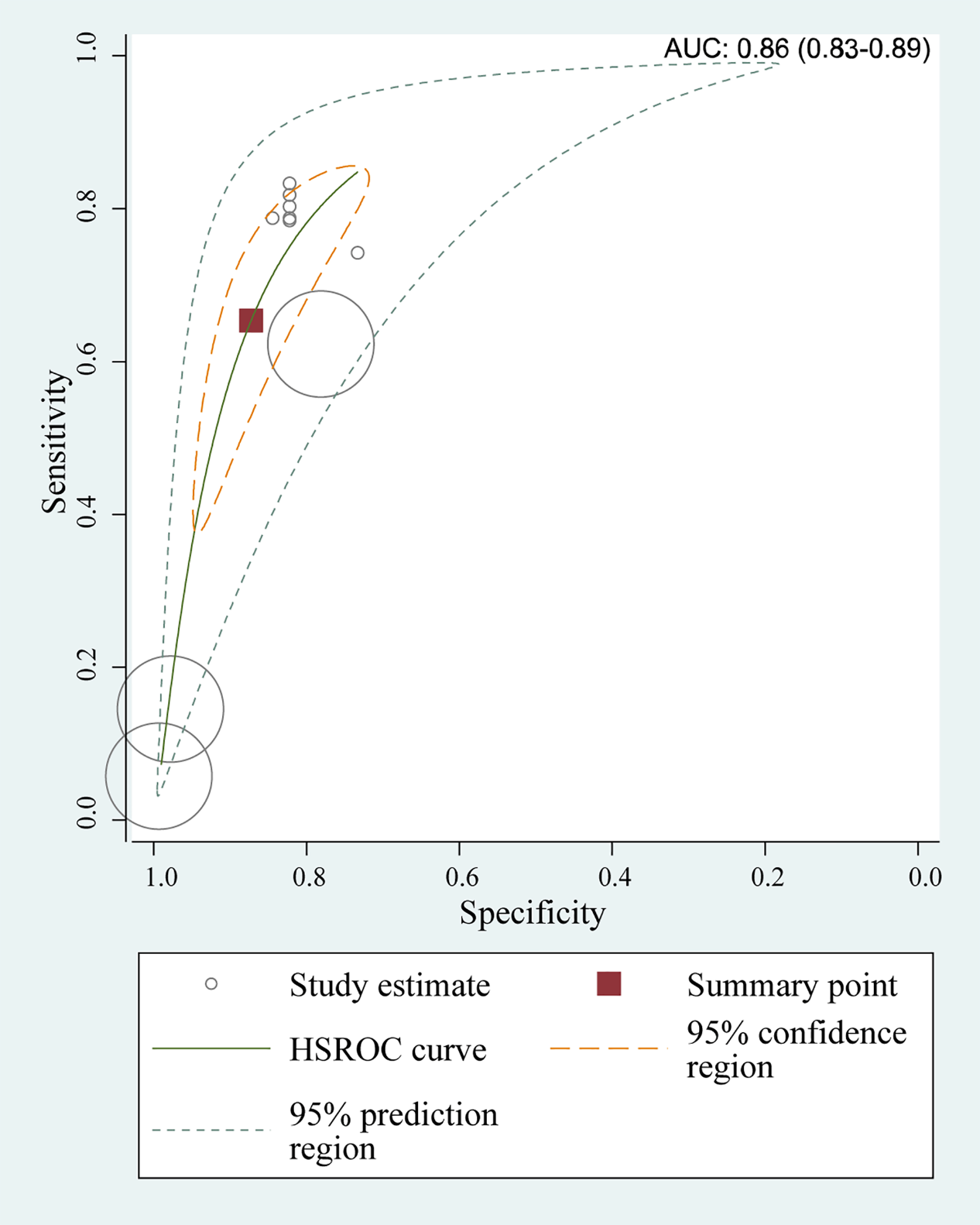


**Figure S7. HSROC curve for Asian group with AUC of 0.82.**


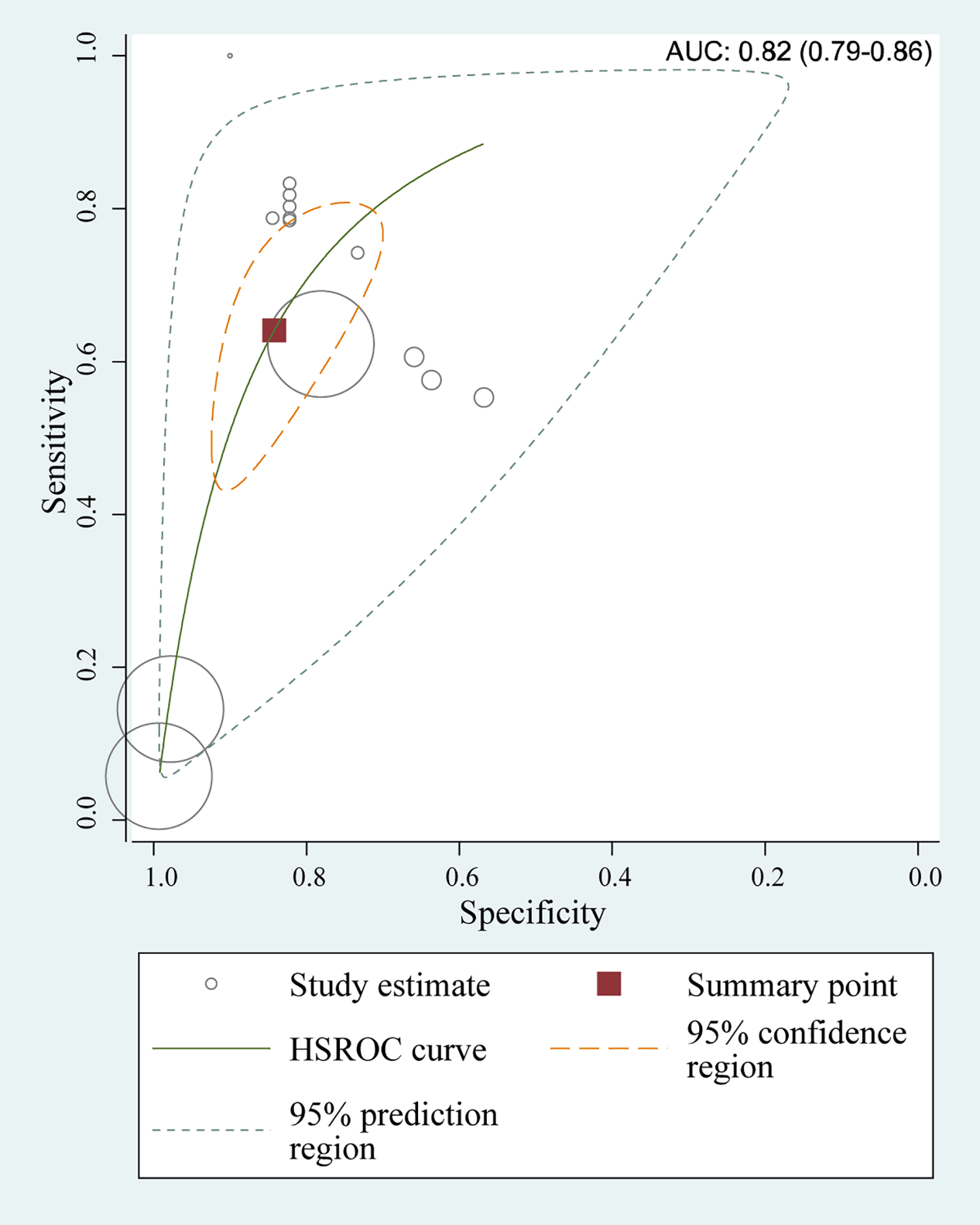


**Figure S8. HSROC curve for non-Asian group with AUC of 0.78.**


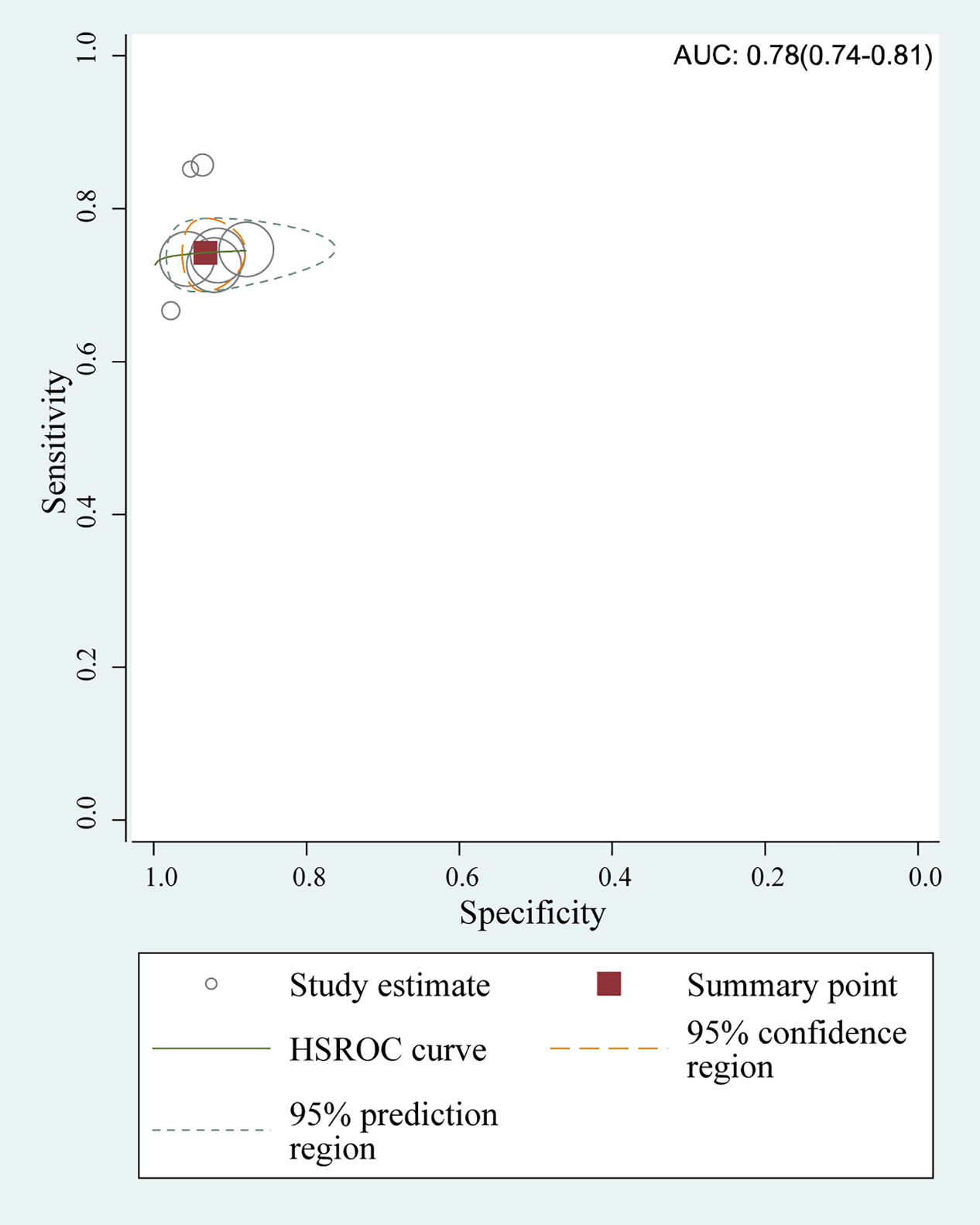

Supplement: Supplementary file 3 — Additional file 3: Figure S5. HSROC curve for subgroup used renal biopsy pathology as a predictor. Figure S6. HSROC curve for subgroup did not use renal biopsy pathology as a predictor. Figure S7. HSROC curve for Asian group. Figure S8. HSROC curve for non-Asian group. [file 12911_2022_1951_MOESM3_ESM.docx]
